# Longitudinal Metagenomic Analysis of Hospital Air Identifies Clinically Relevant Microbes

**DOI:** 10.1371/journal.pone.0160124

**Published:** 2016-08-02

**Authors:** Paula King, Long K. Pham, Shannon Waltz, Dan Sphar, Robert T. Yamamoto, Douglas Conrad, Randy Taplitz, Francesca Torriani, R. Allyn Forsyth

**Affiliations:** 1 FLIR Systems, Inc., La Jolla, California, United States of America; 2 Singlera Genomics, Inc., La Jolla, California, United States of America; 3 Department of Biology, San Diego State University, San Diego, California, United States of America; 4 Zova Systems, LLC, San Diego, California, United States of America; 5 Department of Medicine, Division of Pulmonary Medicine, UC San Diego Health System, San Diego, California, United States of America; 6 Department of Medicine, Division of Infectious Diseases and Infection Prevention and Clinical Epidemiology Unit, UC San Diego Health System, San Diego, California, United States of America; Cornell University, UNITED STATES

## Abstract

We describe the sampling of sixty-three uncultured hospital air samples collected over a six-month period and analysis using shotgun metagenomic sequencing. Our primary goals were to determine the longitudinal metagenomic variability of this environment, identify and characterize genomes of potential pathogens and determine whether they are atypical to the hospital airborne metagenome. Air samples were collected from eight locations which included patient wards, the main lobby and outside. The resulting DNA libraries produced 972 million sequences representing 51 gigabases. Hierarchical clustering of samples by the most abundant 50 microbial orders generated three major nodes which primarily clustered by type of location. Because the indoor locations were longitudinally consistent, episodic relative increases in microbial genomic signatures related to the opportunistic pathogens *Aspergillus*, *Penicillium* and *Stenotrophomonas* were identified as outliers at specific locations. Further analysis of microbial reads specific for *Stenotrophomonas maltophilia* indicated homology to a sequenced multi-drug resistant clinical strain and we observed broad sequence coverage of resistance genes. We demonstrate that a shotgun metagenomic sequencing approach can be used to characterize the resistance determinants of pathogen genomes that are uncharacteristic for an otherwise consistent hospital air microbial metagenomic profile.

## Introduction

Potentially pathogenic aerosolized microorganisms can disperse great distances by air and remain viable [1–3] which raises the possibility of remote infection without contact to the source [4–6]. Modern hospital environmental and administrative controls protect vulnerable patient populations from such risks by maintaining stringent airflow systems and isolating patients with airborne infectious diseases [3]. Despite these controls, there were an estimated 721,000 healthcare-associated infections (HAIs) among acute care facilities in the United States in 2011 [7]. Prospectively detecting potential airborne pathogens and resistance determinants could be an adjunctive measure in a comprehensive approach to reduce HAIs [8].

Culture-independent (CI) studies have used 16S rRNA sequencing to survey the diversity of bacterial genera in indoor air [9–12]. Alternatively, shotgun metagenomic sequencing (SMS) approaches define microbial diversity from the analysis of the relative abundances of sequence reads specific for species and even strains [13–16]. Metagenomic sequencing allows the alignment of millions of identified reads to reconstruct genomes [17] or study the resistome [18] and metabolic signatures [19] which adds valuable information to the study of indoor microbiomes and the effect on the inhabitants.

Microbial metagenomic profiles have been documented in the infant human gut [17, 20], in a large built environment [21], on a city-wide scale [22] and shown to transfer with inhabitants to new homes [23]. Our objective was to longitudinally sample air for six-months in an acute care hospital and use SMS to evaluate the relative abundance of microbial specific reads in order to profile the microbial metagenomes for the sampled locations. By collecting over 60 samples at several indoor locations we anticipated the ability to establish metagenomic profiles and detect unusual increases in read abundance for pathogen genomes. Furthermore, we hoped that SMS could allow us to characterize resistance determinants.

## Materials and Methods

### Sample locations

Air samples were collected from eight locations at a hospital affiliated with the UC San Diego Health System. Outdoor air was collected on the hospital roof. Indoor sampling locations included five areas of a three-floor main lobby and two return air ducts (“ducts”). Air collections occurred on the top two floors of the lobby and included the waiting areas and corridors. Air enters the lobby through floor air supply registers. Exhaust and return air registers in the lobby are located above the third floor to achieve unidirectional airflow from the first to third floor. For each duct sample, we simultaneously collected air from multiple patient rooms via a sampling port installed in the duct prior to reconditioning. One duct location (D1) was at least 6 meters downstream from seven patient rooms of a medical-surgical unit. For the other duct location (D2), air was sampled from a twenty-seven room unit dedicated to severely immunocompromised patients, which included D2 offices and corridors, and was at least 12 meters from the closest patient room. The lobby and patient units of D1 and D2 have fully contained air handling systems, each with tandem air handling units (AHUs). The lobby AHUs contain minimum efficiency reporting value (MERV) 8 and 10 rated filters and have a combined design airflow rate of 41,200 cfm with the required ≥6 air changes per hour (ACH) for an acute care facility built before 1993 [24]. The D1 and D2 AHUs contain MERV 8, 13 and 14 rated filters and outside air passes through pre-filters prior to entering the D2 air handling room. The D1 and D2 unit AHUs maintain ≥6 ACH with a combined return air flow of 11,200 cfm. The temperature and relative humidity of the sampled locations are maintained at 21°C and 40%, respectively.

### Sample collection

Air was sampled using an ACD-200 dry filter air sampler (InnovaPrep, Drexel, MO) for one to nine days, during a six-month period. The ACD-200 is suitable for sampling outdoors or indoors and allows for relatively quiet operation (64 dB). Collection occurs with sterile 52 mm electret collection filter units provided with the Rapid Elution Kit (InnovaPrep) constructed of electrostatically charged dielectric polymer fibers [25, 26] commonly used in HVAC filters. The filter achieves increased collection efficiency of particles, including allergens and microbes, 0.1 μm to 10 μm in size with minimal pressure drops allowing faster sampling rates and extended sampling periods [27, 28], consistent with our goal of continuous monitoring. Sampling occurred at a manufacturer calibrated flow rate of 100 liters per minute (Lpm). Collection occurred at a height of 53–86 cm above the floor in the lobby and 104 cm above the roof. Sampling from the ducts utilized a stainless steel sampling probe (S1 Fig) connected to a custom designed ACD-200 lid (InnovaPrep) with a conductive silicon tube (TSI, Inc., Shoreview, MN) to reduce the accumulation of static charge and minimize particulate sedimentation and deposition on the inner wall of the tube during sampling. Computational fluid dynamic modeling studies using SOLIDWORKS Flow Simulation (Dassault Systèmes, Waltham, MA) estimated the transmission efficiencies of 1–5 μm particles (e.g. bacteria, micro droplets, dust) through the conductive tube.

Once sampling was complete, with gloved hands, the sterile sample cup was inverted over the collection filter, tightly sealed, covered with the sample cup lid and the entire assembly was inserted into a zippered plastic bag. The filter assembly was transported at ambient temperature to a BSL-2 research laboratory. Between samples, all ACD-200 components immediately upstream or in contact with the filter unit were thoroughly wiped with 70% isopropanol.

### Sample processing and DNA extraction

Filters were eluted in a Type-II biosafety cabinet according to manufacturer instructions using 0.075% Tween 20, 25 mM Tris elution foam supplied with the Rapid Elution Kit. A 1 ml aliquot was archived at -20°C. Approximately 5 ml remained and was frozen at -20°C for later batch processing. At the time of DNA extraction, the filter eluate was thawed at room temperature, mixed by pipetting and filtered through a sterile syringe assembly consisting of a 25 mm, 0.2 μm membrane (Whatman #7402–002, GE Healthcare Life Sciences, Pittsburgh, PA) housed in a Swin-Lok 25 mm membrane holder (Whatman #420200–25). The membrane was aseptically removed and DNA was isolated using the PowerWater DNA Isolation Kit (MO BIO Laboratories, Carlsbad, CA) protocol with the modification of elution with two subsequent 50 μl volumes of 60°C elution buffer. Free DNA in the liquid filtrate that passed through the membrane was precipitated with 0.3 M ammonium acetate and 100% ethanol at -20°C for a minimum of 16 hours followed by centrifugation at 15,000 x g for 30 minutes at 4°C. The DNA pellet was washed twice with 70% ethanol, dried and resuspended using the respective membrane PowerWater kit eluate, resulting in the final DNA sample. A flow chart details the air sample processing and DNA isolation (S2 Fig). A similarly processed blank air collection filter served as a negative control.

### DNA measurement and qPCR analysis

Total DNA concentrations were determined using the Qubit dsDNA HS Assay Kit and the Qubit 2.0 Fluorometer (LifeTechnologies, Carlsbad, CA). Quantitative real-time PCR was used to determine the relative abundance of bacterial and fungal DNA per sample (S2 Table).

### NGS library preparation and sequencing

The Nextera XT DNA Sample Preparation Kit (Illumina, San Diego, CA) was used to prepare barcoded DNA fragment libraries. Prior to library preparation with the DNA from hospital air samples we evaluated metagenomic variability below the recommended 1 ng kit input with triplicate 0.04 ng and 0.004 ng Nextera XT DNA libraries using DNA from an indoor air sample. Depending on the hospital location, libraries from 0.01 ng (duct) or 0.1 ng (outside, lobby) DNA inputs were prepared and submitted for sequencing. Samples with low DNA yields that could not be quantified by Qubit assay had libraries prepared with the maximum recommended kit input volume of 5 μl. Depending on the input DNA concentration, the tagmentation products were amplified using 15–19 cycles and DNA was purified with Agencourt AMPure XP beads (Beckman Coulter, Indianapolis, IN). Library quality and size distribution was determined with the 2100 BioAnalyzer and High Sensitivity DNA Analysis Kit (Agilent Technologies, Santa Clara, CA). Libraries were diluted to 2 nM for sequencing and 10–13 libraries were pooled and sequenced per lane on a single flow cell. Manufacturer instructions were followed for the Illumina HiSeq 2500 Rapid Run or High Output modes to obtain 50 nucleotide read lengths. Sequencing was performed by the IGM Genomics Center, University of California, San Diego (La Jolla, CA).

### Sequence analysis

Illumina raw data sets were analyzed after barcode removal as described previously [29, 30] and microbial taxa were identified with the automated ZovaSeq pipeline (Zova Systems, LLC, San Diego, CA). A sequence read that was found to occur uniquely within a given bacterial, fungal, viral, archaeal or protozoal taxon defined by the NCBI taxonomy database [31, 32] was assigned to that taxon and designated a “microbial ID read”. All assigned microbial ID reads were binned according to higher taxonomy (e.g., order, family) based on the April 2015 NCBI GenBank Release 207.0. Subsequent analyses involved only the microbial ID reads. Human reads were mapped using Bowtie 1.0.0 with parameters allowing two mismatches in a 28 bp seed region. All sequences related to this study have been archived in the NCBI Sequence Read Archive under BioProject number PRJNA287928.

### Dendrogram and statistical analysis

Reads specific for each NCBI microbial order (or families for viruses, when NCBI does not identify an order) were tallied for all samples and normalized to the total microbial ID reads for each sample. The fifty most abundant orders were determined by the average read counts per order across all samples (the remaining orders or families were summed into “Other” counts). The read counts were rank ordered and entered into Cluster 3.0 [33] for hierarchical clustering using the Euclidean distance similarity metric with default filter settings. The dendrogram was drawn using TreeView [34]. A two-tailed t-test for unequal variances was conducted to determine whether dendrogram nodes were statistically different.

## Results

### Airborne DNA recovery

Sixty-three air samples were collected between April and October 2014 with a sampling duration of one to nine days, in order to obtain sufficient DNA yields for our NGS approach, and included forty-two samples from five main lobby locations, nineteen samples from two ducts and two from the hospital roof. Accessing the ducts required the use of a sampling probe and conductive silicone tube with a calculated transmission efficiency of 50–90% for 1–5 μm particles. The ducts had the lowest DNA concentrations (0.1–26.5 pg/m^3^), likely a result of the high volume of air flow in the duct and sampling at least 6 meters from the most proximal patient room. Differences were observed in the density of genomic DNA among these locations, as is reflected in the extracted airborne DNA concentrations (Table 1). Among the indoor samples, the lobby had the highest DNA concentration and also the broadest DNA concentration range (0–101.2 pg/m^3^). In contrast, the ducts had the lowest overall DNA concentration range (0.1–26.5 pg/m^3^). No bacterial or fungal genomes were detected in the negative control sample using universal gram-negative and gram-positive bacterial and universal fungal qPCR assays.

**Table 1:** Air samples collected (n = 63).

Location	n	Collection Time (days)	Total Air Volume (m3)	Total DNA Concentration (pg/m3)
Roof	2	3.0 (3.0–3.1)	437 (428–446)	16.1 (4.6–27.6)
Lobby	42	4.2 (1.0–8.9)	594 (104–1275)	14.3 (0.0–101.2)
L1	18	4.4 (1.0–7.0)	602 (104–1014)	10.7 (2.4–36.3)
L2	6	4.8 (4.1–5.0)	693 (594–764)	18.4 (4.6–49.5)
L3	2	4.9 (4.8–5.0)	729 (695–764)	1.7 (0.0–3.4)
L4	13	4.0 (1.8–8.9)	575 (264–1275)	18.6 (3.5–101.2)
L5	3	3.0 (1.8–5.2)	431 (265–745)	18.1 (7.6–25.9)
Return Air Duct	19	4.8 (4.1–5.0)	530 (225–1171)	3.3 (0.1–26.5)^a^
D1	9	4.5 (2.0–8.8)	578 (268–1171)	0.5 (0.1–1.2)^a^
D2	10	6.5 (4.1–7.1)	487 (225–723)	5.9 (0.2–26.5)^a^

Data is shown as average (range).

^a^Total DNA from the duct samples was typically below the limit of detection for the Qubit HS assay. Sample DNA concentrations were approximated with microbial qPCR assays (S1 Table).

### NGS library and sequencing results

Validation of low DNA inputs into the metagenomic protocol was executed with triplicate 0.04 ng and 0.004 ng Nextera XT DNA libraries. The results demonstrated similar representations of identified microbial taxa which justified our low DNA input NGS pipeline (S3 Fig). The range of library fragment sizes for the 63 samples collected was 300–1100 bp and the range of library yields was 0.3–20 ng/μl. Two lobby samples failed to produce quality libraries and were not sequenced. The remaining 61 samples averaged 16 million total 50 base reads (range: 6.4–37.8 million) with sequence quality scores from 34.85 to 38.02. The average total reads for the roof, lobby and duct locations were similar.

### Metagenomic analysis

**Fig 1:**
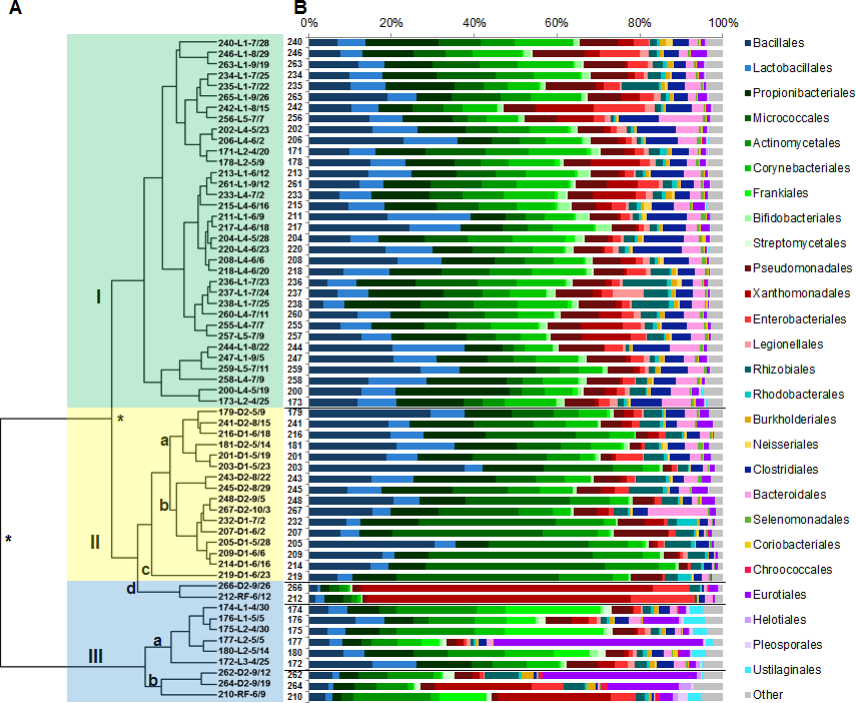
Location-specific metagenomic profiles are disturbed by increases in relative abundances of genomic reads. (A) Dendrogram of the air samples with three nodes: (I) main lobby (L) samples shaded green, (IIa-c) duct (D) samples shaded yellow and (IId,III) outlier samples shaded blue. Significant differences between nodes (*p*<0.05) are noted by an asterisk. (B) Corresponding microbial metagenomic profiles represented by the percent distribution of normalized (to 1 M) read counts for 26 orders with >1% microbial abundance. Color schemes represent orders grouped by class or phylum. “Other” is represented by the percent sum of the remaining 24 orders.

Our analysis of the 61 samples produced an average of 31% assigned reads and 69% unassigned reads. Among the assigned reads, microbial ID reads accounted for 31% (range: 14–85%) while human reads represented 69% (range: 15–86%) (Table 2). All duct samples had a higher average percent of microbial ID reads than all lobby samples, 37% vs. 27%, respectively. We identified an average of 562 unique microbial species per sample when including single microbial ID reads. We identified the fifty most abundant orders with greater than one percent relative read abundance. The rank ordered average percent of microbial ID read counts for the fifty orders (S2 Table) was used to construct a dendrogram which showed the samples segregated into three major nodes (Fig 1A). Eighty-five percent (34/40) of the lobby samples clustered into node I with similar microbial order profiles (Fig 1B) and node II contained eighty-nine percent (17/19) of the duct samples. Nodes I and II combined accounted for eight-four percent (51/61) of all collected samples. Two-tailed t-tests assuming unequal variances from weighted average read counts indicated significant differences between nodes I and II (t(20) = 9.13, *p =* 1.4x10^-8^) and between nodes I and II combined compared to node III (t(18) = -4.26, *p* = 4.8x10^-4^).

**Table 2:** NGS summary of air samples (n = 61).

Location	n	Total Reads	Assigned Reads		
			Total	Microbial^a^	Human
Roof	2	16.8 ± 3.2	2.6 ± 3.3	1.1 ± 1.3 (43)	1.5 ± 2.0 (57)
Lobby	40	16.3 ± 6.0	4.4 ± 2.7	1.2 ± 0.6 (27)	3.2 ± 2.2 (73)
L1	17	16.5 ± 4.7	4.7 ± 2.6	1.3 ± 0.5 (28)	3.4 ± 2.2 (72)
L2	6	19.5 ± 9.3	3.2 ± 1.1	1.2 ± 0.7 (38)	2.0 ± 0.5 (62)
L3	1	14.4	3.4	1.0 (29)	2.4 (71)
L4	13	16.2 ± 5.7	4.8 ± 3.4	1.2 ± 0.8 (24)	3.7 ± 2.8 (76)
L5	3	10.2 ± 1.1	3.9 ± 1.9	0.8 ± 0.3 (20)	3.1 ± 1.6 (80)
Return Air Duct	19	15.1 ± 7.4	6.4 ± 4.5	2.4 ± 0.9 (37)	4.0 ± 4.0 (63)
D1	9	12.3 ± 3.0	5.9 ± 2.4	2.5 ± 0.7 (42)	3.5 ± 2.3 (58)
D2	10	17.5 ± 9.4	6.8 ± 5.9	2.3 ± 1.1 (34)	4.5 ± 5.2 (66)

Reads are expressed in millions as average ± standard deviation. The average percent of assigned microbial or human reads is shown in parentheses.

^a^Microbial reads include: Bacteria, fungi, virus, protozoa, and archaea.

Among all node I lobby samples, 73% of the microbial ID reads were assigned to eight bacterial orders with greater than 5% average relative abundance (*Bacillales*, *Lactobacillales*, *Propionibacteriales*, *Micrococcales*, *Actinomycetales*, *Corynebacteriales*, *Pseudomonadales*, *Xanthomonadales*) representing three classes (*Bacilli*, *Actinobacteria*, *Gammaproteobacteria*) from three phyla (*Firmicutes*, *Actinobacteria*, *Proteobacteria*) (S2 Table). These same eight orders comprised 81% of microbial ID reads in node II samples which were predominantly from the ducts. However, only 54% of the microbial ID reads for samples in node III were assigned to the same orders, largely due to an influx of *Frankiales*, *Xanthomonodales* or *Eurotiales* reads (S2 Table).

The eleven samples that clustered separately in subnodes IId, IIIa and IIIb were determined to be uncharacteristic for their location-specific genomic profiles. Six of the samples had increased relative read abundances for the order *Frankiales* attributable to increased *Geodermatophilus obscurus* reads. Subnode IId included a June roof sample (212) and September D2 sample (266) and both had a majority of microbial ID reads map to *Xanthomonadales*, specifically to *Stenotrophomonas maltophilia* (63.8% and 70.5%, respectively).

**Fig 2:**
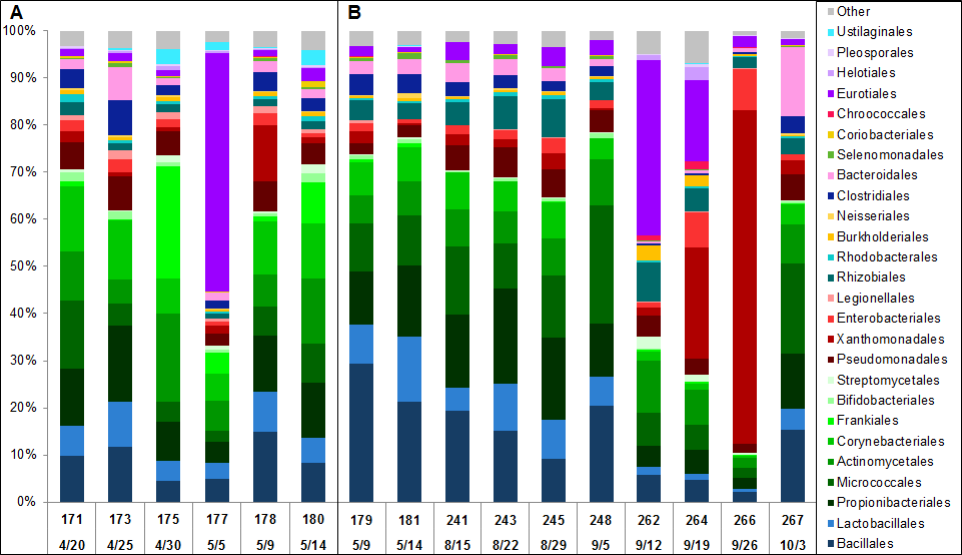
Longitudinal sampling identifies changes in location profiles. Microbial metagenomic profiles representing the percent distribution of normalized (to 1 M) read counts for 26 microbial orders with >1% abundance aid in the identification of samples with unusual profiles (A) in lobby L2 between April 30 –May 5 and (B) in duct D2 between September 5–26. Sample numbers and collection dates are indicated. Color schemes represent orders grouped by class or phylum. “Other” is represented by the percent sum of the remaining 24 orders.

Continuous five-day samples in lobby areas over four weeks showed a fairly stable profile with the exception of samples 176 and 177 which clustered with the outliers in node III and not with the lobby samples in node I. *Eurotiales* microbial ID reads in node I had a standard deviation just under 1%. Sample 177 was collected from location L2 and had a relative increase in *Eurotiales* reads from an overall average of 3% to 50% (Fig 2A), mainly due to increased *Aspergillus niger* reads, followed by return to the typical metagenomic profile for this location. Sample 176 was simultaneously collected from location L1 approximately 15 meters away and also had a relative increase in reads specific for *A*. *niger* (S2 Table).

We also examined the D2 samples (Fig 2B) in a time course. The metagenomic profile of D2 was represented by two five-day samples in May (179, 181) and four seven-day samples from August to early September (241, 243, 245 and 248) which clustered in subnodes IIa and IIb. The D2 profile dramatically changed during three consecutive weeks in September when a large relative increase in *Eurotiales* specific microbial ID reads was followed by a relative increase in *Xanthomonadales* specific reads. Sample 262 (September 5–12) had 37.2% of reads specific for *Eurotiales*, which included a mixture of *Penicillium chrysogenum* and *Aspergillus fumigatus* reads. The following week in sample 264 (Sept. 12–19), *Xanthomonadales* specific reads increased to 23.6%, primarily from reads specific for *S*. *maltophilia*. *S*. *maltophilia* specific reads then increased to represent the majority of the microbial reads (70.5%) in sample 266 (Sept. 19–26). Samples 262, 264 and 266 clustered in subnode IId or node III. Sample 267 (Sept. 26 –October 3), the last D2 sample collected, clustered in subnode IIb with the majority of D2 samples, representing a return to the initial D2 profile.

**Fig 3:**
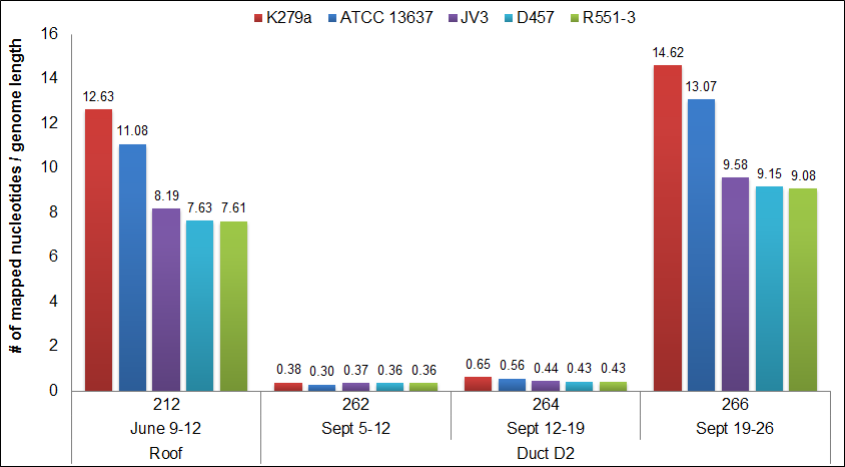
Depth of mapped read coverage to fully sequenced *S*. *maltophilia* genomes.

**Fig 4:**
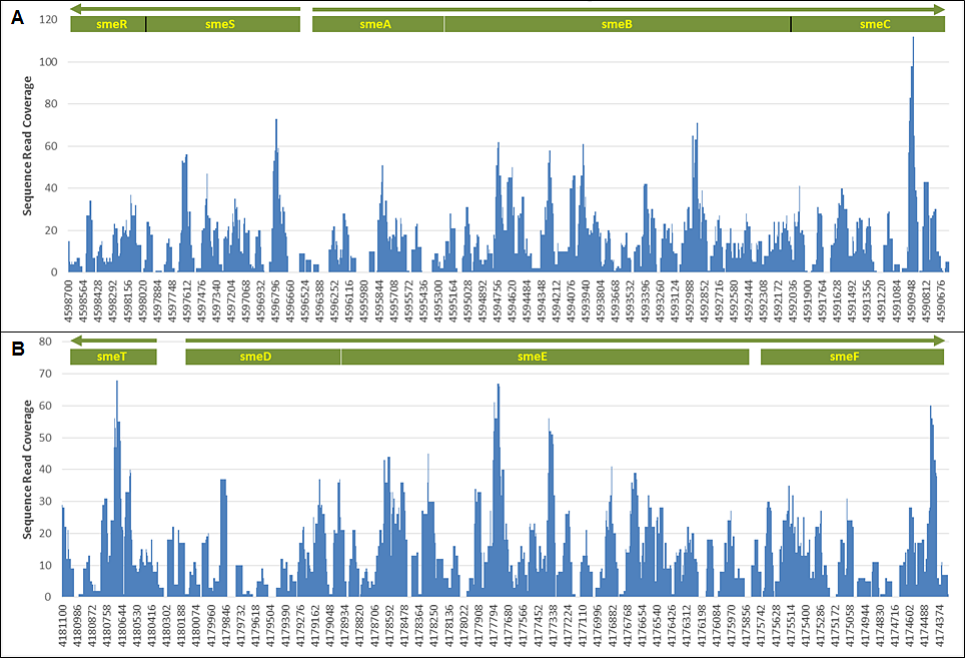
*S*. *maltophilia* K279a efflux pump operon read coverage depth. (A) smeSF-smeABC. (B) smeT-smeDEF. The x-axis labels indicate the position on the K279a genome. The white lines represent intergenic gaps and the black lines represent gene distinction.

*S*. *maltophilia* reads for the three-day roof sample 212 (June 9–12) and D2 samples 262, 264 and 266 were mapped to each of the five *S*. *maltophilia* complete genomes in NCBI (Fig 3). The *S*. *maltophilia* strain with the closest sequence identity to these samples, as defined by depth of sequence coverage, was the multi-drug resistant clinical strain K279a [35]. Moreover, sample 266 sequences covered the majority of K279a resistance nodulation division efflux pump operons *sme*ABC and *sme*DEF and the respective regulator genes *sme*SR and *sme*T [36]. The *sme*SR-*sme*ABC and *sme*T-*sme*DEF operons were 86.5% and 88.5% sequenced with an average depth of coverage of 14.6-fold and 13.2-fold, respectively (Fig 4). Efflux pump overexpression can confer high-level antibiotic resistance to multiple antibiotics and the sequenced regions of sample 266 shared 100% nucleotide identity with K279a with the exception of *sme*D in which we detected a single nucleotide polymorphism (SNP) that likely confers a conservative valine to alanine substitution at amino acid position 213.

## Discussion

We describe an application of SMS to study the microbial genomes of indoor air using an approach similar to recent outdoor air studies [13, 16]. To facilitate our initial understanding of hospital air we examined only the microbial identifying sequences and the human genome as previously described [29, 30]. The percent of assigned and unassigned NGS reads in our study was similar to previous metagenomic NGS studies [12, 22]. We demonstrated the relative stability of microbial genomic profiles in hospital air but did not examine the viability or ultimate sources of the microbial variations detected.

Air duct sampling is technically challenging but is desirable as an unobtrusive resource with respect to patients. Our tests show an expected lower efficiency of sampling from ducts, lower average DNA recoveries, and metagenomic profiles which are statistically distinct from nearby lobbies. Only two roof samples were collected as a simple reference and we detected increased reads for a particular *S*. *maltophilia* strain in roof sample 212 that matched reads from D2 samples 262, 264 and 266 collected three months later. Although these samples were collected months apart, there is the potential for indoor increases in microbial content to be attributed to the introduction of outdoor air [37]. Also, five of the six node III lobby samples had increased reads for the Actinobacteria *Geodermatophilus obscurus*, commonly found in samples of rock and desert sand [38], which lends support to a potential outdoor source.

The observed microbiota of an indoor environment is only as diverse as the occupants and the hygienic standards. The metagenomic profiles we established included the fifty most abundant microbial orders and tracked the major constituents in this hospital’s air. Just eight microbial orders accounted for more than 75% of the relative reads in the majority of lobby and duct samples. Included among the predominant species detected were human commensals belonging to the orders *Bacillales*, *Propionibacteriales*, *Corynebacteriales*, *Enterobacteriales*, in addition to constituents from various ecological habitats, including soil (*Pseudomonadales*) and leaf (*Xanthomonadales*) [9, 39, 40]. The phyla Firmicutes, Actinobacteria and Proteobacteria were highly represented as reported in other built environments [21, 23, 40, 41], including a hospital [12].

For this study, our immediate interest was to determine the stability of the microbial metagenome. For all samples, the standard deviation of relative read abundances for each order was typically less than 5% which indicated a consistent, low-level presence in the air, yet increased for four orders, including *Xanthomonodales* and *Eurotiales*. Such baseline metagenomic profiles allowed us to identify *S*. *maltophilia*, *A*. *niger*, *A*. *fumigatus and P*. *chrysogenum* as outliers during our longitudinal sampling. In contrast, a high degree of microbiome fluctuation was reported from hourly samples collected in a New York train station during the course of a day [22]. Our work supports the ability to identify outlier events in daily to weekly increments based on a longitudinal baseline in an environment such as a hospital where efforts are made to maintain clean and relatively static environments.

Hierarchical clustering of the relative read counts of the top fifty microbial orders for each sample resulted in distinct nodes clustered primarily by location. Variability in the abundance of assigned microbial reads at a location altered the rank order and implied a change in the airborne metagenome profile. We observed the metagenomes of duct D2 and other indoor locations had occasional fluctuations yet returned to location associated profiles. Lobby samples 176 and 177 simultaneously collected 15 meters apart had increased read counts for *Aspergillus* and *Penicillium*. Yooseph, et. al. reported a higher number of relative metagenomic reads for *Aspergillus* and *Penicillium* inside a hospital compared to outside the hospital and inside a home [12].

Three samples from duct D2 (262, 264 and 266) yielded high DNA concentrations, more comparable to the lobby and roof air, and had altered D2 profiles due to specific increased read abundances for *P*. *chrysogenum*, *A*. *fumigatus* and *S*. *maltophilia*; the detected microbial ID reads are of interest since these microbes are considered opportunistic pathogens among immunocompromised patient populations [42–44]. Tringe, et. al. reported a greater proportion of Proteobacteria with a higher abundance of metagenomic reads related to *S*. *maltophilia* from indoor HVAC duct air samples compared to water and soil samples [21]. Other environmental hospital studies reported increases in viable *Penicillium*, *Aspergillus* or *S*. *maltophilia* [44–46] attributable to indoor reservoirs or environmental disturbances. Disruption of a microbial profile can be influenced by the diversity of occupants and their health status or hospital activities [47, 48], which we plan to evaluate in future studies.

Although the majority of *S*. *maltophilia* microbial ID reads from D2 samples 262, 264 and 266 shared closest identity to the complete genome sequence of the multi-drug resistant clinical strain K279a, the abundance of reads dramatically increased in sample 266. The deeper sequence coverage that resulted from sample 266 reads allowed us to identify resistance determinants and analyze SNPs. This SMS downstream approach has application for use in clinical infection prevention and epidemiologic studies [18, 49, 50].

We envision improvements to monitoring indoor metagenomes will result from key technical and informatics advancements. Recent NGS enhancements such as clutter mitigation and genomic enrichment technologies [29, 30, 51] increase the depth of genome coverage and can facilitate the detection of polymorphisms associated with antimicrobial resistance. The potential for more refined airborne strain recognition is expected to expand with more accessible and robust NGS platforms [52, 53], reduced sequencing costs [54] and more complete sequences of microbial genomes.

Our work indicates that longitudinal SMS can facilitate the identification of airborne pathogen genomes and resistance genotypes that are outliers compared to an otherwise stable metagenomic profile. The relevance of our findings within the hospital environment has yet to be determined but this data set should prove valuable to those interested in the dynamics of the hospital air metagenome. The metagenomic profiles of human microbiota are becoming increasingly characterized, and growing data suggests imbalances could lead to disease states [55]. We envision that more comprehensive metagenomic profiles of indoor environments will facilitate the identification of imbalances that result from the introduction of new or pathogenic microbes. In a hospital environment, this ability could translate to identifying associations with clinical or environmental events, leading to a better understanding of the spread of healthcare-associated infections, enhanced preventative measures and, ultimately, improved health outcomes.

## Conclusion

We applied SMS for the study of the hospital air microbiota during a six-month period. The microbial relative read abundances of the locations sampled were relatively stable which allowed for the creation of metagenomic profiles by location and facilitated the identification of relative increases in genomes of opportunistic pathogens. We chronicled sporadic indoor air metagenomic fluctuations and identified the species and strain identity of non-cultured airborne constituents and SNPs in antibiotic resistance genes. We conclude that continuously monitoring indoor air using SMS has potential as a near real-time versatile approach for environmental metagenomic surveillance. Ultimately, the utility of monitoring hospital air metagenomes requires further study and will rely on the rapid association of events that contribute to increases in HAI pathogens to apply appropriate interventions and improve health outcomes.

## Supporting Information

S1 Fig In-duct sampling probe installation in relation to the direction of air flow. (PDF)

S2 Fig Flow chart for air sample processing. (PDF)

S3 Fig Pairwise comparison of triplicate 0.04 ng and 0.004 ng SMS libraries produced from the same source DNA. (PDF)

S1 Table Real-time quantitative PCR assays. (XLSX)

S2 Table Sample rank average percent of the most abundant 50 microbial orders grouped by higher taxonomy. (PDF)
