# Correction: Longitudinal Metagenomic Analysis of Hospital Air Identifies Clinically Relevant Microbes

**DOI:** 10.1371/journal.pone.0169376

**Published:** 2016-12-28

**Authors:** Paula King, Long K. Pham, Shannon Waltz, Dan Sphar, Robert T. Yamamoto, Douglas Conrad, Randy Taplitz, Francesca Torriani, R. Allyn Forsyth

The Materials and Methods section does not describe the ZovaSeq pipeline used to analyze the data in sufficient detail. The attached Supporting Information file provides additional information about the version of ZovaSeq used for King et al. 2016 [1], which is currently available for use from Zova Systems (contact robert.yamamoto@zovasystems.com; also see https://lnkd.in/gxkRCVV). ZovaSeq has since been updated and further optimized.

## Supporting Information

S1 File King et al. supplemental—ZovaSeq method. (PDF)
