# Supplementary figures and images for: Longitudinal Metagenomic Analysis of Hospital Air Identifies Clinically Relevant Microbes
Source: PLoS One. 2016 Aug 2;11(8):e0160124. doi: 10.1371/journal.pone.0160124 (PMC4970769; doi:10.1371/journal.pone.0160124)

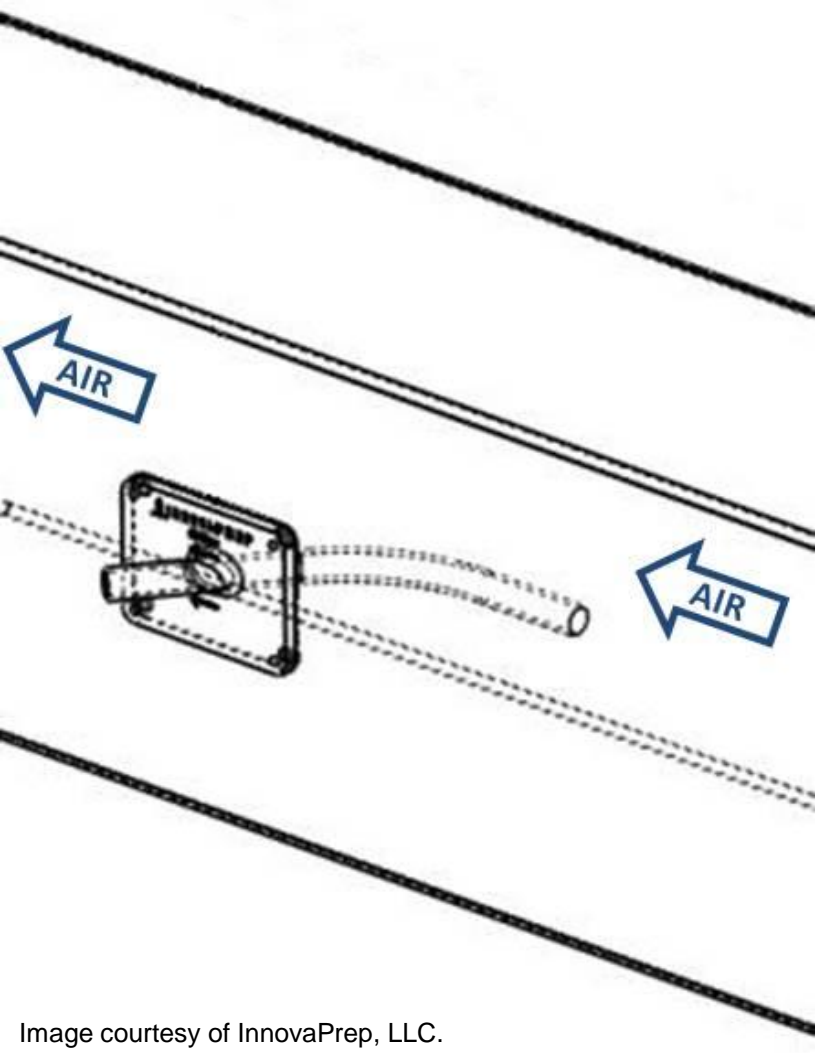

Image courtesy of InnovaPrep, LLC.

Supplement: S1 Fig — (PDF) [file pone.0160124.s001.pdf]

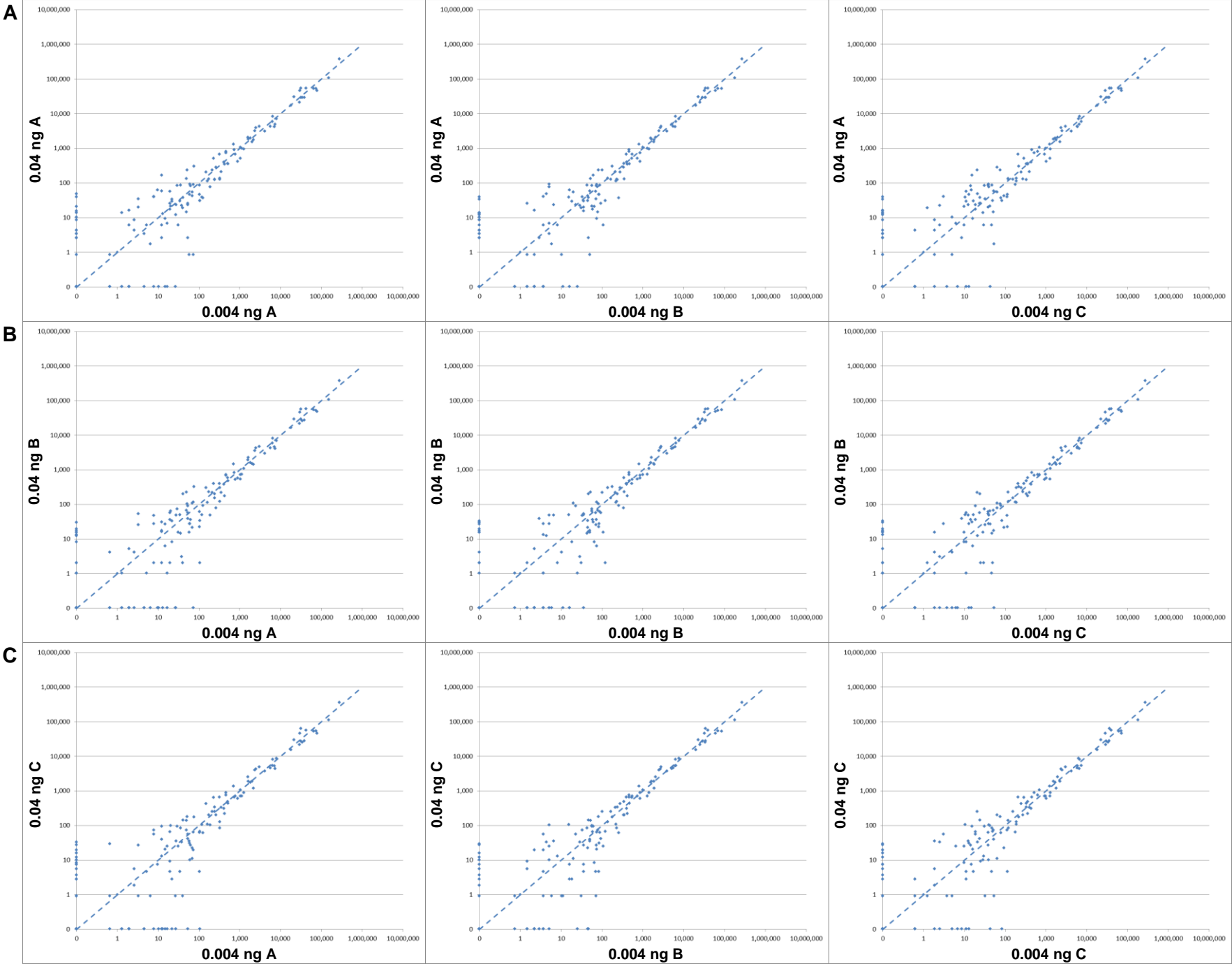

Supplement: S3 Fig — (PDF) [file pone.0160124.s003.pdf]
