# Supplementary material for: Longitudinal Metagenomic Analysis of Hospital Air Identifies Clinically Relevant Microbes
Source: PLoS One. 2016 Aug 2;11(8):e0160124. doi: 10.1371/journal.pone.0160124 (PMC4970769; doi:10.1371/journal.pone.0160124)
Supplement: S2 Fig — (PDF) [file pone.0160124.s002.pdf]

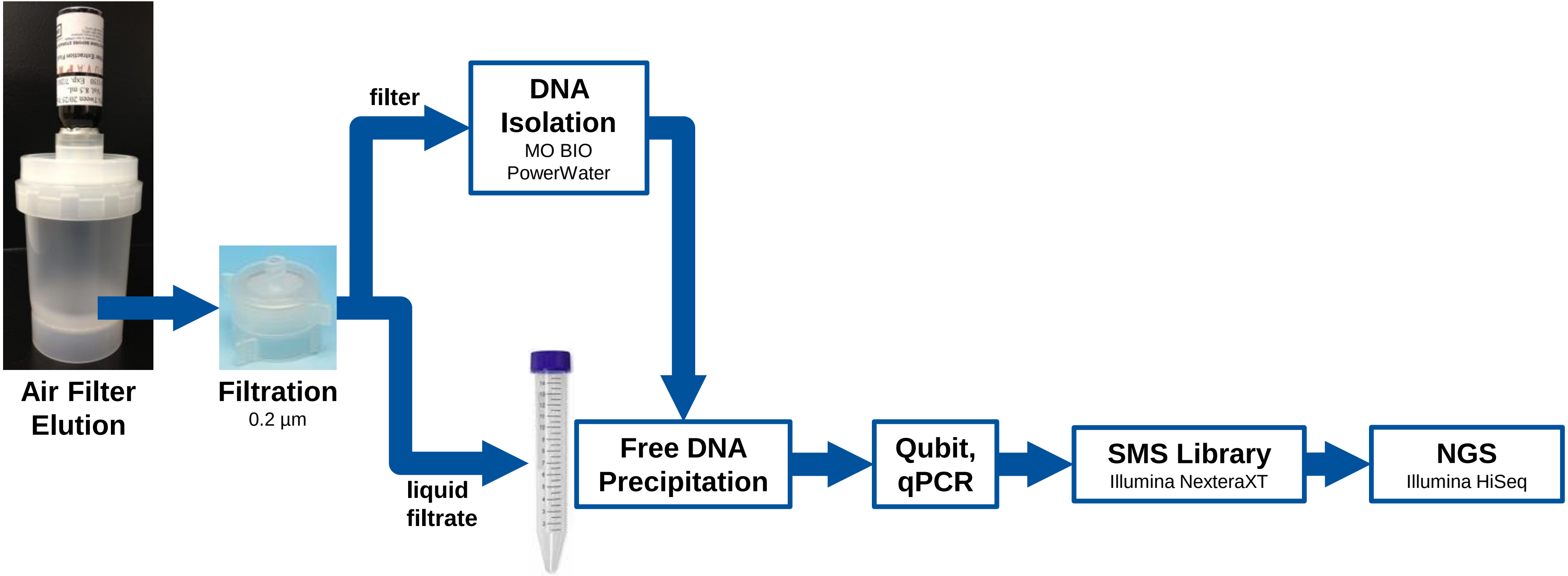
Air Filter Elution
Filtration
0.2 µm
filter
DNA Isolation
MO BIO PowerWater
liquid filtrate
Free DNA Precipitation
Qubit, qPCR
SMS Library
Illumina NexteraXT
NGS
Illumina HiSeq
