# Supplementary material for: Longitudinal Metagenomic Analysis of Hospital Air Identifies Clinically Relevant Microbes
Source: PLoS One. 2016 Aug 2;11(8):e0160124. doi: 10.1371/journal.pone.0160124 (PMC4970769; doi:10.1371/journal.pone.0160124)
Supplement: S2 Table — (PDF) [file pone.0160124.s005.pdf]

Highest relative percent abundance — **Lowest relative percent abundance**

PHYLUM (or higher)	Firmicutes					Actinobacteria										
CLASS	Bacilli		Clostridia	Negativicutes	Erysipelotrichia	Actinobacteria										Coriobacteriia
ORDER	Bacillales	Lactobacillales	Clostridiales	Selenomonadales	Erysipelotrichales	Propionibacteriales	Micrococcales	Actinomycetales	Corynebacteriales	Frankiales	Bifidobacteriales	Streptomycetales	Pseudonocardiales	Streptosporangiales	Micromonosporales	Coriobacteriales
240-L1-7/28	6.96	6.69	3.87	0.39	0.10	17.65	10.28	8.07	14.14	0.19	1.18	0.35	0.09	0.09	0.06	0.29
246-L1-8/29	7.74	5.35	3.54	0.36	0.06	12.27	7.80	6.49	11.72	0.45	1.84	0.50	0.33	0.07	0.08	0.27
263-L1-9/19	11.99	6.24	2.93	0.38	0.07	11.59	11.59	9.01	13.29	0.57	1.69	0.50	0.18	0.09	0.10	0.29
234-L1-7/25	9.98	7.83	3.36	0.52	0.09	12.99	14.36	9.19	11.04	0.53	1.59	0.50	0.13	0.06	0.11	0.38
235-L1-7/22	10.20	8.40	4.27	0.46	0.13	16.69	7.02	5.84	7.48	0.33	1.05	0.32	0.08	0.09	0.05	0.24
265-L1-9/26	19.10	6.88	3.48	0.31	0.07	8.49	11.96	7.15	12.08	0.29	0.92	0.41	0.09	0.18	0.08	0.21
242-L1-8/15	10.40	6.43	6.17	0.45	0.08	8.29	7.45	4.61	8.16	0.30	1.20	0.21	0.08	0.02	0.03	0.24
256-L5-7/7	14.64	7.93	9.79	0.74	0.15	12.15	3.70	4.45	7.66	0.17	1.42	0.12	0.05	0.01	0.03	0.21
202-L4-5/23	15.40	10.79	9.35	0.60	0.22	11.61	7.84	7.62	9.31	0.82	1.40	0.30	0.09	0.07	0.06	0.36
206-L4-6/2	22.90	12.96	7.74	0.90	0.13	8.43	6.68	6.23	8.54	0.42	1.73	0.27	0.08	0.04	0.06	0.44
171-L2-4/20	9.76	6.36	4.08	0.32	0.08	12.19	14.40	10.35	13.96	1.07	1.86	0.52	0.17	0.13	0.10	0.38
178-L2-5/9	14.90	8.47	4.11	0.57	0.13	11.91	6.24	6.85	11.14	1.02	0.77	0.40	0.12	0.07	0.09	0.27
213-L1-6/12	14.45	10.34	8.06	0.63	0.19	10.90	9.44	7.21	12.00	0.68	2.67	0.42	0.11	0.05	0.06	0.45
261-L1-9/12	12.40	5.58	3.08	0.36	0.07	11.37	12.44	8.33	12.71	0.46	0.82	0.50	0.15	0.08	0.08	0.25
233-L4-7/2	7.47	7.78	3.81	0.63	0.07	14.04	7.86	9.92	12.34	0.67	1.93	0.42	0.13	0.10	0.12	0.27
215-L4-6/16	9.69	8.55	5.29	0.47	0.06	12.73	10.15	9.01	9.38	0.57	2.96	0.36	0.17	0.20	0.08	0.27
211-L1-6/9	18.98	20.21	9.60	1.13	0.17	8.40	4.84	4.55	6.91	0.47	3.36	0.21	0.07	0.03	0.04	0.27
217-L4-6/18	24.28	12.50	5.63	0.65	0.11	10.15	6.00	6.04	9.91	0.36	3.70	0.21	0.07	0.03	0.05	0.25
204-L4-5/28	10.00	6.87	9.62	0.76	0.11	11.20	10.28	10.91	12.88	2.18	1.91	0.38	0.13	0.06	0.10	1.01
220-L4-6/23	18.62	11.30	11.96	0.69	0.34	9.68	7.68	6.76	7.50	0.64	1.63	0.29	0.08	0.03	0.09	0.21
208-L4-6/6	21.48	10.66	5.97	0.88	0.11	12.73	7.28	6.96	7.21	0.54	1.46	0.35	0.10	0.06	0.05	0.25
218-L4-6/20	8.33	11.26	4.23	0.64	0.06	12.49	11.62	10.24	12.79	0.45	1.24	0.47	0.12	0.04	0.10	0.27
236-L1-7/23	4.56	6.91	2.67	0.43	0.05	14.41	14.84	9.42	11.76	0.36	1.14	0.38	0.11	0.04	0.08	0.24
237-L1-7/24	7.05	7.43	4.14	0.49	0.07	18.05	9.36	6.56	8.71	0.28	1.92	0.27	0.07	0.03	0.07	0.34
238-L1-7/25	3.66	5.01	2.87	0.40	0.04	18.25	12.95	10.51	12.14	0.88	1.27	0.50	0.15	0.07	0.13	0.33
260-L4-7/11	11.70	8.14	4.67	0.55	0.11	14.05	8.67	7.92	8.60	0.32	1.26	0.26	0.07	0.05	0.06	0.30
255-L4-7/7	7.61	7.63	5.99	0.52	0.12	12.84	7.54	8.43	11.28	0.48	1.51	0.31	0.08	0.03	0.06	0.34
257-L5-7/9	12.66	7.43	4.66	0.74	0.17	18.10	6.86	6.05	6.15	0.14	0.85	0.17	0.07	0.04	0.10	0.24
244-L1-8/22	20.24	17.40	8.86	0.94	0.30	9.12	2.76	2.74	6.61	0.11	1.20	0.12	0.04	0.03	0.01	0.23
247-L1-9/5	20.52	10.32	4.63	0.58	0.18	12.26	6.38	5.44	10.01	0.28	1.64	0.19	0.07	0.04	0.06	0.25
259-L5-7/11	26.97	9.57	6.68	0.77	0.05	18.23	5.88	4.45	5.75	0.14	1.15	0.15	0.03	0.05	0.04	0.11
258-L4-7/9	14.56	13.99	5.43	0.83	0.17	19.25	3.81	4.86	8.55	0.15	1.81	0.07	0.01	0.03	0.01	0.41
200-L4-5/19	12.86	8.08	5.01	0.40	0.21	27.71	2.74	5.69	6.64	1.30	0.81	0.60	0.13	0.13	0.01	0.13
173-L2-4/25	11.78	9.44	7.42	0.92	0.15	16.19	4.69	5.13	12.50	0.21	1.70	0.22	0.06	0.04	0.03	0.37
179-D2-5/9	29.42	8.33	4.52	0.58	0.17	11.18	10.17	6.02	6.89	0.72	0.76	0.19	0.04	0.07	0.02	0.22
241-D2-8/15	19.36	4.95	2.79	0.56	0.06	15.56	14.44	7.82	7.75	0.05	0.36	0.17	0.05	0.04	0.02	0.13
216-D1-6/18	19.84	7.98	3.11	0.66	0.04	14.92	21.74	8.30	5.80	0.05	0.36	0.06	0.01	0.02	0.01	0.12
181-D2-5/14	21.30	13.95	4.04	1.20	0.22	15.06	10.58	7.14	7.25	0.90	0.89	0.36	0.08	0.18	0.03	0.23
201-D1-5/19	18.88	7.54	5.01	0.54	0.08	13.09	18.01	7.81	3.59	0.50	0.69	0.36	0.06	0.06	0.03	0.14
203-D1-5/23	37.63	4.48	2.95	0.35	0.03	14.75	16.57	7.16	3.92	0.22	0.73	0.16	0.03	0.03	0.02	0.18
243-D2-8/22	15.27	9.98	2.83	0.95	0.10	20.02	9.59	6.90	6.24	0.07	0.57	0.28	0.04	0.05	0.02	0.15
245-D2-8/29	9.32	8.28	2.07	0.43	0.08	17.24	13.17	7.86	7.79	0.11	0.75	0.22	0.14	0.31	0.02	0.13
248-D2-9/5	20.45	6.22	2.18	0.58	0.09	11.19	25.05	9.90	4.34	0.10	1.00	0.10	0.03	0.08	0.01	0.29
267-D2-10/3	15.47	4.30	3.66	0.33	0.24	11.69	19.20	8.32	4.28	0.11	0.46	0.20	0.05	0.08	0.03	0.15
232-D1-7/2	9.03	3.54	1.72	0.33	0.03	14.04	32.98	11.59	2.90	0.17	0.27	0.10	0.01	0.03	0.02	0.06
207-D1-6/2	7.80	4.51	1.26	0.41	0.02	8.05	36.24	13.19	2.77	0.42	0.46	0.23	0.09	0.33	0.02	0.11
205-D1-5/28	30.50	5.03	3.05	0.37	0.11	8.03	24.00	9.38	3.70	0.68	0.68	0.14	0.04	0.02	0.04	0.12
209-D1-6/6	17.79	3.01	1.27	0.19	0.02	8.08	39.77	12.08	4.00	0.09	0.71	0.16	0.02	0.02	0.02	0.04
214-D1-6/16	14.89	4.41	1.85	0.21	0.02	8.98	43.11	13.25	3.88	0.06	0.73	0.11	0.02	0.04	0.02	0.10
219-D1-6/23	7.08	3.51	1.93	0.47	0.03	10.57	39.32	13.04	3.42	0.41	0.37	0.15	0.02	0.01	0.02	0.07
266-D2-9/26	2.19	0.62	0.47	0.07	0.02	2.30	2.28	2.05	0.48	0.08	0.16	0.44	0.04	0.06	0.02	0.01
212-RF-6/12	1.68	2.12	1.55	0.10	0.05	4.74	1.92	1.43	0.67	0.19	0.08	0.11	0.05	0.01	0.01	0.01
174-L1-4/30	4.68	4.69	2.71	0.23	0.09	7.67	4.11	19.42	7.12	22.90	0.77	1.84	0.46	0.36	0.19	0.16
176-L1-5/5	11.21	7.06	3.47	0.39	0.09	7.00	3.79	11.03	7.79	6.98	0.78	1.57	0.37	0.34	0.14	0.19
175-L2-4/30	4.47	4.36	2.24	0.24	0.04	8.19	4.24	18.79	7.37	23.83	0.75	1.54	0.40	0.25	0.15	0.16
177-L2-5/5	4.99	3.31	1.88	0.13	0.05	4.52	2.38	6.27	5.87	4.41	0.70	0.79	0.25	0.24	0.07	0.10
180-L2-5/14	8.35	5.23	2.75	0.42	0.05	11.67	8.35	13.75	11.85	8.55	1.93	2.01	0.44	0.38	0.15	1.21
172-L3-4/25	15.33	10.66	4.12	0.66	0.12	13.41	5.09	8.12	7.89	0.46	0.88	0.53	0.12	0.13	0.06	0.21
262-D2-9/12	5.83	1.59	0.51	0.12	0.03	4.54	7.03	11.08	1.84	0.42	0.25	2.58	0.31	0.50	0.16	0.07
264-D2-9/19	4.80	1.29	0.35	0.14	0.02	5.09	5.31	7.29	1.37	0.42	0.11	1.39	0.36	0.27	0.06	0.03
210-RF-6/9	4.09	1.63	0.58	0.21	0.09	2.13	3.07	18.49	2.24	11.24	0.10	1.16	0.67	0.29	0.32	0.03

PHYLUM (or higher)	Proteobacteria															
CLASS	Gammaproteobacteria							Alphaproteobacteria					Betaproteobacteria		Deltaproteobacteria	Epsilonproteobacteria
ORDER	Pseudomonadales	Xanthomonadales	Enterobacteriales	Legionellales	Pasteurellales	Alteromonadales	Aeromonadales	Rhizobiales	Rhodobacterales	Caulobacterales	Sphingomonadales	Rhodospirillales	Burkholderiales	Neisseriales	Myxococcales	Campylobacterales
240-L1-7/28	9.07	4.00	3.49	0.23	0.52	0.12	0.08	1.85	0.54	0.40	0.20	0.08	1.43	1.74	0.01	0.03
246-L1-8/29	12.52	3.55	9.75	2.10	0.28	0.08	0.05	1.92	0.70	0.35	0.38	0.09	0.75	0.37	0.10	0.02
263-L1-9/19	9.84	0.83	4.73	1.14	0.44	0.18	0.07	2.51	1.01	0.38	0.47	0.16	1.46	0.26	0.06	0.06
234-L1-7/25	9.06	1.73	2.13	0.94	0.45	0.14	0.05	3.20	1.18	0.63	0.46	0.16	1.01	0.49	0.04	0.05
235-L1-7/22	12.10	1.86	3.88	0.55	0.26	0.26	0.11	8.74	0.50	0.69	0.34	0.14	1.10	0.59	0.03	0.13
265-L1-9/26	8.14	4.62	3.22	1.68	0.16	0.21	0.07	1.40	0.76	0.26	0.32	0.12	0.75	0.28	0.04	0.07
242-L1-8/15	7.67	14.09	12.30	2.53	0.20	0.10	0.03	1.29	0.50	0.20	0.21	0.06	0.57	0.29	0.03	0.07
256-L5-7/7	7.66	8.80	2.89	1.45	0.32	0.19	0.08	0.64	0.38	0.20	0.20	0.03	0.42	0.30	0.03	0.04
202-L4-5/23	6.23	1.72	1.43	2.34	0.26	0.11	0.05	1.02	0.56	0.17	0.21	0.07	0.59	0.23	0.04	0.02
206-L4-6/2	6.58	1.88	1.36	1.28	0.50	0.15	0.05	0.86	0.42	0.15	0.18	0.07	0.50	0.30	0.03	0.05
171-L2-4/20	5.88	2.36	2.25	1.14	0.17	0.03	0.03	2.76	1.71	0.35	0.46	0.15	0.88	0.30	0.19	0.02
178-L2-5/9	6.31	11.86	2.59	1.43	0.22	0.14	0.04	1.58	0.66	0.26	0.31	0.11	0.84	0.22	0.13	0.07
213-L1-6/12	6.67	1.43	2.29	0.46	0.51	0.11	0.04	1.63	0.79	0.23	0.30	0.11	0.63	0.49	0.03	0.03
261-L1-9/12	7.28	7.67	5.08	0.93	0.22	0.12	0.05	2.15	1.18	0.33	0.36	0.12	0.75	0.27	0.03	0.05
233-L4-7/2	6.27	10.30	2.49	1.48	0.40	0.22	0.09	2.22	1.06	0.37	0.48	0.12	1.52	0.54	0.08	0.03
215-L4-6/16	5.98	3.83	3.32	0.74	0.93	0.10	0.02	2.06	0.81	0.31	0.30	0.11	0.68	2.03	0.14	0.06
211-L1-6/9	6.80	0.69	2.04	0.87	0.38	0.07	0.05	1.47	0.37	0.15	0.19	0.07	0.58	0.85	0.02	0.09
217-L4-6/18	5.91	0.70	1.09	1.50	0.34	0.11	0.03	1.74	0.46	0.16	0.20	0.08	0.64	0.31	0.04	0.17
204-L4-5/28	5.54	1.27	1.98	0.58	0.32	0.13	0.07	2.69	0.89	0.21	0.33	0.12	0.81	0.62	0.05	0.04
220-L4-6/23	6.67	0.61	2.09	1.46	0.47	0.11	0.04	1.39	0.94	0.30	0.20	0.07	0.56	0.32	0.04	0.06
208-L4-6/6	6.59	3.66	1.81	1.73	0.25	0.14	0.06	1.12	0.68	0.22	0.24	0.09	0.61	0.87	0.04	0.18
218-L4-6/20	7.27	0.75	4.41	1.39	0.31	0.10	0.04	4.38	0.63	0.25	0.30	0.21	0.85	0.45	0.03	0.09
236-L1-7/23	7.84	1.53	2.06	0.56	0.27	0.16	0.07	10.72	0.91	0.72	0.52	0.16	1.50	1.17	0.07	0.04
237-L1-7/24	8.85	2.25	2.78	7.44	0.26	0.20	0.06	5.78	0.67	0.49	0.33	0.11	0.93	0.36	0.03	0.05
238-L1-7/25	8.96	1.37	2.11	0.31	0.17	0.12	0.06	9.12	1.04	0.56	0.40	0.14	1.02	0.44	0.05	0.11
260-L4-7/11	9.02	5.78	2.83	1.76	0.22	0.45	0.22	3.77	0.68	0.41	0.36	0.11	0.92	0.26	0.05	0.08
255-L4-7/7	8.01	10.20	4.44	0.83	0.19	0.12	0.08	2.04	0.90	0.36	0.37	0.09	0.74	0.51	0.03	0.03
257-L5-7/9	7.64	11.77	3.69	0.25	0.34	0.08	0.08	3.96	0.52	0.25	0.23	0.06	1.07	0.35	0.01	0.05
244-L1-8/22	10.95	0.34	4.35	0.71	0.46	0.38	0.05	0.72	0.20	0.12	0.17	0.05	0.56	0.24	0.01	0.10
247-L1-9/5	9.61	1.19	3.02	1.55	0.26	0.16	0.10	1.20	0.84	0.19	0.34	0.10	1.20	0.35	0.02	0.14
259-L5-7/11	6.17	1.39	1.30	0.97	0.24	0.02	0.04	1.41	0.34	0.15	0.23	0.03	0.90	0.19	0.00	0.08
258-L4-7/9	8.01	0.91	2.73	0.35	0.57	0.35	0.06	2.56	0.33	0.14	0.09	0.02	0.84	0.49	0.08	0.11
200-L4-5/19	4.97	3.04	1.88	1.27	0.72	0.05	0.02	3.83	0.30	0.08	0.15	0.06	0.66	0.16	0.06	0.04
173-L2-4/25	7.23	0.87	2.74	1.85	0.45	0.06	0.04	1.63	0.59	0.22	0.33	0.07	0.58	0.55	0.03	0.03
179-D2-5/9	2.45	2.47	1.70	0.70	0.34	0.02	0.01	4.17	0.52	0.06	0.06	0.05	0.30	0.23	0.01	0.02
241-D2-8/15	5.15	2.47	1.76	0.16	0.15	0.07	0.10	4.82	0.73	0.17	0.13	0.04	0.37	0.26	0.02	0.02
216-D1-6/18	2.82	2.65	1.76	0.08	0.62	0.01	0.01	3.86	0.33	0.08	0.05	0.02	0.25	0.58	0.00	0.03
181-D2-5/14	2.61	0.34	0.76	0.15	0.52	0.03	0.03	3.33	0.34	0.06	0.08	0.04	0.64	1.21	0.01	0.03
201-D1-5/19	2.66	0.98	6.64	0.08	0.35	0.01	0.01	5.01	0.53	0.09	0.10	0.04	0.33	0.39	0.01	0.02
203-D1-5/23	1.74	0.20	1.31	0.21	0.23	0.01	0.00	2.50	0.24	0.06	0.06	0.03	0.19	0.33	0.01	0.01
243-D2-8/22	6.46	1.56	1.91	0.25	0.28	0.04	0.02	7.06	0.75	0.64	0.25	0.05	0.54	0.40	0.01	0.03
245-D2-8/29	5.81	3.46	3.28	0.08	0.17	0.03	0.04	8.19	0.81	0.73	0.28	0.10	0.69	0.21	0.02	0.02
248-D2-9/5	4.76	0.40	1.63	0.07	0.16	0.02	0.02	3.94	0.47	0.10	0.13	0.03	0.46	0.23	0.01	0.02
267-D2-10/3	5.59	2.89	1.24	0.04	0.12	0.03	0.05	3.31	0.49	0.14	0.12	0.04	0.30	0.33	0.01	0.01
232-D1-7/2	6.53	4.55	0.92	0.20	0.13	0.05	0.01	2.18	4.67	0.31	0.08	0.20	0.72	0.19	0.02	0.02
207-D1-6/2	12.99	0.20	1.76	0.12	0.06	0.02	0.03	4.18	0.61	0.14	0.14	0.05	0.28	0.38	0.01	0.01
205-D1-5/28	1.30	0.90	1.48	0.17	0.10	0.02	0.00	6.20	0.95	0.06	0.04	0.06	0.19	0.25	0.01	0.00
209-D1-6/6	3.50	0.46	0.74	1.22	0.04	0.00	0.00	4.14	0.65	0.13	0.04	0.03	0.16	0.13	0.00	0.00
214-D1-6/16	1.79	0.28	0.71	0.03	0.06	0.00	0.00	2.45	0.51	0.06	0.04	0.03	0.15	0.15	0.01	0.01
219-D1-6/23	7.22	0.50	0.46	0.03	0.17	0.03	0.06	3.14	3.08	0.13	0.07	0.10	0.26	0.39	0.01	0.01
266-D2-9/26	1.88	70.54	8.90	0.02	0.03	0.04	0.03	2.49	0.16	0.07	0.06	0.03	0.43	0.02	0.02	0.00
212-RF-6/12	0.94	63.81	15.54	0.01	0.39	0.03	0.00	0.40	0.09	0.03	0.03	0.01	0.17	0.20	0.01	0.02
174-L1-4/30	4.86	0.54	1.50	0.54	0.17	0.07	0.04	1.91	0.73	0.24	0.44	0.24	1.03	0.18	0.17	0.03
176-L1-5/5	6.39	3.97	1.93	1.09	0.16	0.08	0.04	1.41	0.66	0.29	0.41	0.16	1.05	0.21	0.15	0.06
175-L2-4/30	5.13	0.76	1.73	1.45	0.16	0.06	0.03	1.84	0.60	0.26	0.43	0.22	0.91	0.30	0.21	0.04
177-L2-5/5	2.62	1.49	0.90	0.80	0.08	0.04	0.02	0.89	0.40	0.16	0.23	0.10	0.54	0.10	0.09	0.02
180-L2-5/14	4.31	1.30	1.01	0.84	0.21	0.49	0.05	1.63	0.97	0.22	0.39	0.16	0.84	0.31	0.12	0.02
172-L3-4/25	7.46	4.10	3.02	1.74	0.40	0.07	0.04	3.01	0.76	0.55	0.53	0.13	1.23	0.49	0.02	0.09
262-D2-9/12	4.37	1.72	1.08	0.14	0.04	0.08	0.02	8.30	0.57	0.37	0.41	0.19	3.05	0.07	0.06	0.01
264-D2-9/19	3.30	23.59	7.60	0.08	0.06	0.09	0.07	4.95	0.42	0.14	0.34	0.12	2.23	0.10	0.06	0.01
210-RF-6/9	1.41	27.39	5.93	0.16	0.02	0.35	0.01	2.15	0.85	0.21	0.39	0.12	0.71	0.03	0.32	0.01

PHYLUM (or higher)	Ascomycota							Bacteroidetes		Basidiomycota			Fusobacteria	Cyanobacteria			Verrucomicrobia	Viruses	
CLASS	Eurotiomycetes	Dothideomycetes	Leotiomycetes	Saccharomycetes		Sordariomycetes		Bacteroidia	Flavobacteriia	Ustilaginomycetes	Malasseziomycetes	Agaricomycetes	Fusobacteriia				Verrucomicrobiae	dsDNA viruses	
ORDER	Eurotiales	Pleosporales	Helotiales	Saccharomycetales	mitosporic Saccharomycetales	Sordariales	Hypocreales	Bacteroidales	Flavobacteriales	Ustilaginales	Malasseziales	Polyporales	Fusobacteriales	Chroococcales	Nostocales	Oscillatoriales	Verrucomicrobiales	Caudovirales	Other[a]
240-L1-7/28	0.98	0.17	0.46	0.14	0.22	0.02	0.04	2.16	0.15	0.05	0.35	0.36	0.03	0.01	0.04	0.10	0.02	0.06	0.42
246-L1-8/29	3.74	0.13	0.22	0.20	0.06	0.03	0.02	2.28	0.07	0.01	0.17	0.00	0.05	0.04	0.05	0.50	0.05	0.04	0.45
263-L1-9/19	1.46	0.18	0.37	0.16	0.08	0.04	0.04	1.93	0.12	0.02	0.24	0.01	0.06	0.09	0.06	0.25	0.02	0.05	0.73
234-L1-7/25	1.05	0.24	0.32	0.24	0.04	0.03	0.04	2.30	0.11	0.02	0.22	0.23	0.07	0.03	0.03	0.02	0.03	0.06	0.52
235-L1-7/22	0.29	0.09	0.24	0.26	0.06	0.03	0.03	3.88	0.16	0.02	0.16	0.07	0.17	0.03	0.05	0.01	0.02	0.10	0.41
265-L1-9/26	2.22	0.24	0.21	0.23	0.13	0.03	0.06	1.82	0.09	0.01	0.28	0.02	0.06	0.05	0.08	0.03	0.02	0.06	0.56
242-L1-8/15	1.19	0.15	0.20	0.18	0.07	0.02	0.04	2.88	0.12	0.01	0.18	0.01	0.10	0.02	0.06	0.02	0.02	0.05	0.45
256-L5-7/7	0.66	0.08	0.21	0.15	0.03	0.01	0.04	10.69	0.17	0.02	0.15	0.04	0.19	0.01	0.09	0.03	0.10	0.06	0.37
202-L4-5/23	1.08	0.44	0.12	0.32	0.13	0.02	0.02	5.58	0.14	0.20	0.23	0.00	0.07	0.02	0.09	0.02	0.03	0.05	0.57
206-L4-6/2	0.91	0.36	0.16	0.31	0.18	0.02	0.02	4.61	0.18	0.15	0.21	0.01	0.20	0.02	0.08	0.01	0.02	0.08	0.55
171-L2-4/20	1.34	0.25	0.40	0.25	0.05	0.03	0.02	2.07	0.13	0.13	0.16	0.01	0.04	0.03	0.04	0.02	0.02	0.04	0.51
178-L2-5/9	1.34	0.35	0.14	0.31	0.08	0.02	0.02	2.27	0.15	0.18	0.16	0.02	0.12	0.07	0.05	0.01	0.02	0.07	0.77
213-L1-6/12	0.56	0.21	0.02	0.25	0.09	0.06	0.02	4.11	0.12	0.04	0.19	0.00	0.20	0.03	0.07	0.01	0.04	0.04	0.52
261-L1-9/12	1.46	0.12	0.01	0.14	0.05	0.05	0.03	1.76	0.10	0.01	0.20	0.01	0.07	0.03	0.06	0.02	0.02	0.04	0.56
233-L4-7/2	0.70	0.12	0.03	0.22	0.06	0.02	0.02	2.58	0.12	0.02	0.17	0.01	0.09	0.01	0.04	0.04	0.01	0.06	0.43
215-L4-6/16	2.90	0.23	0.02	0.16	0.07	0.03	0.02	3.92	0.11	0.05	0.19	0.00	0.09	0.02	0.05	0.02	0.17	0.04	0.51
211-L1-6/9	0.80	0.20	0.01	0.21	0.08	0.01	0.02	3.56	0.26	0.06	0.12	0.00	0.19	0.02	0.05	0.01	0.03	0.04	0.37
217-L4-6/18	2.22	0.28	0.02	0.25	0.10	0.01	0.02	2.60	0.15	0.09	0.19	0.00	0.12	0.01	0.04	0.01	0.02	0.06	0.39
204-L4-5/28	0.79	0.19	0.02	0.15	0.05	0.01	0.02	3.62	0.13	0.08	0.13	0.00	0.08	0.01	0.05	0.03	0.03	0.03	0.53
220-L4-6/23	0.46	0.28	0.00	0.21	0.09	0.01	0.02	4.69	0.20	0.07	0.18	0.00	0.13	0.01	0.11	0.03	0.03	0.05	0.57
208-L4-6/6	0.35	0.28	0.06	0.31	0.07	0.01	0.02	3.33	0.16	0.09	0.21	0.00	0.13	0.02	0.04	0.07	0.04	0.07	0.41
218-L4-6/20	0.25	0.14	0.05	0.18	0.09	0.01	0.01	2.45	0.17	0.04	0.20	0.00	0.10	0.01	0.02	0.02	0.04	0.05	0.36
236-L1-7/23	0.80	0.07	0.00	0.20	0.07	0.01	0.02	2.06	0.09	0.01	0.12	0.15	0.05	0.06	0.04	0.01	0.02	0.05	0.44
237-L1-7/24	0.48	0.08	0.00	0.10	0.02	0.01	0.03	2.82	0.08	0.03	0.18	0.13	0.05	0.02	0.04	0.01	0.02	0.05	0.42
238-L1-7/25	0.35	0.13	0.01	0.12	0.01	0.03	0.02	3.11	0.08	0.02	0.16	0.16	0.04	0.02	0.02	0.03	0.02	0.05	0.47
260-L4-7/11	0.66	0.12	0.04	0.16	0.06	0.02	0.02	3.93	0.12	0.04	0.18	0.11	0.09	0.01	0.05	0.03	0.04	0.05	0.61
255-L4-7/7	0.66	0.06	0.04	0.14	0.03	0.02	0.02	4.18	0.14	0.01	0.11	0.05	0.09	0.01	0.03	0.01	0.23	0.06	0.39
257-L5-7/9	0.28	0.20	0.07	0.19	0.04	0.04	0.02	3.41	0.13	0.02	0.12	0.03	0.13	0.01	0.06	0.02	0.06	0.09	0.29
244-L1-8/22	0.68	0.13	0.11	0.30	0.18	0.02	0.04	7.11	0.21	0.06	0.16	0.01	0.25	0.01	0.09	0.07	0.03	0.07	0.37
247-L1-9/5	2.07	0.23	0.04	0.21	0.11	0.02	0.05	2.69	0.14	0.04	0.26	0.02	0.16	0.04	0.12	0.10	0.02	0.05	0.54
259-L5-7/11	0.65	0.11	0.08	0.30	0.13	0.03	0.05	3.68	0.08	0.07	0.26	0.00	0.08	0.01	0.18	0.05	0.02	0.01	0.75
258-L4-7/9	0.68	0.10	0.17	0.20	0.25	0.00	0.07	4.93	0.32	0.04	0.21	0.02	0.16	0.01	0.50	0.01	0.21	0.14	0.40
200-L4-5/19	1.76	0.38	0.06	0.32	0.11	0.01	0.02	4.41	0.19	0.65	0.55	0.01	0.32	0.01	0.03	0.01	0.68	0.06	0.66
173-L2-4/25	1.83	0.27	0.36	0.27	0.12	0.02	0.03	6.86	0.23	0.33	0.16	0.04	0.17	0.04	0.06	0.01	0.51	0.07	0.50
179-D2-5/9	2.15	0.05	0.02	0.21	0.71	0.00	0.02	2.88	0.09	0.05	0.59	0.01	0.09	0.05	0.00	0.01	0.32	0.05	0.26
241-D2-8/15	3.75	0.02	0.00	0.16	0.07	0.01	0.01	4.18	0.04	0.01	0.46	0.00	0.04	0.01	0.01	0.00	0.30	0.04	0.32
216-D1-6/18	0.19	0.01	0.05	0.12	0.11	0.00	0.00	2.27	0.06	0.02	0.57	0.00	0.14	0.01	0.00	0.01	0.11	0.03	0.13
181-D2-5/14	1.15	0.06	0.02	0.17	0.09	0.00	0.01	3.13	0.23	0.35	0.41	0.01	0.34	0.01	0.01	0.00	0.09	0.06	0.28
201-D1-5/19	1.61	0.03	0.01	0.20	0.43	0.03	0.01	2.63	0.13	0.18	0.66	0.01	0.12	0.01	0.00	0.00	0.03	0.02	0.28
203-D1-5/23	1.30	0.03	0.01	0.19	0.17	0.02	0.01	1.03	0.05	0.07	0.39	0.01	0.15	0.00	0.00	0.00	0.02	0.02	0.19
243-D2-8/22	2.05	0.02	0.01	0.10	0.07	0.02	0.24	3.25	0.09	0.01	0.37	0.00	0.10	0.01	0.01	0.05	0.04	0.05	0.22
245-D2-8/29	3.95	0.03	0.00	0.19	0.13	0.02	0.26	2.63	0.05	0.00	0.44	0.01	0.03	0.01	0.00	0.00	0.07	0.04	0.33
248-D2-9/5	3.13	0.04	0.06	0.18	0.18	0.01	0.03	1.44	0.06	0.01	0.42	0.00	0.07	0.01	0.01	0.03	0.02	0.03	0.19
267-D2-10/3	1.29	0.02	0.06	0.11	0.03	0.01	0.02	14.58	0.07	0.01	0.23	0.01	0.05	0.01	0.01	0.00	0.02	0.03	0.18
232-D1-7/2	0.41	0.03	0.01	0.14	0.09	0.01	0.00	1.00	0.09	0.01	0.24	0.01	0.14	0.02	0.00	0.01	0.02	0.05	0.15
207-D1-6/2	0.92	0.02	0.00	0.20	0.18	0.01	0.02	0.53	0.05	0.01	0.84	0.00	0.03	0.00	0.00	0.00	0.01	0.01	0.26
205-D1-5/28	0.45	0.02	0.00	0.16	0.07	0.00	0.00	0.32	0.04	0.05	0.93	0.00	0.04	0.01	0.05	0.00	0.01	0.01	0.22
209-D1-6/6	0.48	0.01	0.00	0.17	0.18	0.00	0.00	0.18	0.02	0.01	0.26	0.01	0.02	0.00	0.01	0.00	0.01	0.02	0.08
214-D1-6/16	0.61	0.01	0.00	0.13	0.09	0.00	0.00	0.45	0.03	0.03	0.50	0.00	0.03	0.01	0.01	0.00	0.03	0.02	0.10
219-D1-6/23	0.21	0.01	0.00	0.39	0.12	0.00	0.01	1.64	0.08	0.01	0.34	0.00	0.05	0.02	0.00	0.76	0.04	0.03	0.21
266-D2-9/26	2.19	0.04	0.13	0.01	0.01	0.01	0.02	0.71	0.02	0.00	0.04	0.09	0.01	0.29	0.00	0.00	0.01	0.09	0.29
212-RF-6/12	0.28	0.07	0.20	0.00	0.00	0.00	0.00	1.94	0.03	0.11	0.01	0.00	0.04	0.05	0.00	0.00	0.06	0.01	0.79
174-L1-4/30	1.72	0.73	0.35	0.17	0.04	0.12	0.05	1.61	0.09	3.10	0.09	0.04	0.04	0.15	0.47	0.02	0.02	0.03	1.07
176-L1-5/5	8.61	0.78	0.48	0.19	0.06	0.13	0.07	2.70	0.16	5.03	0.08	0.06	0.09	0.14	0.06	0.02	0.02	0.04	1.02
175-L2-4/30	1.30	0.71	0.38	0.12	0.02	0.06	0.04	1.36	0.09	3.35	0.11	0.03	0.04	0.15	0.04	0.04	0.02	0.03	0.95
177-L2-5/5	50.60	0.35	0.18	0.11	0.04	0.06	0.05	1.55	0.06	1.84	0.08	0.02	0.02	0.05	0.04	0.00	0.02	0.02	0.49
180-L2-5/14	2.77	0.30	0.27	0.15	0.04	0.07	0.03	1.95	0.09	3.30	0.18	0.03	0.05	0.04	0.02	0.01	0.01	0.03	0.71
172-L3-4/25	1.52	0.47	1.02	0.20	0.08	0.14	0.04	2.70	0.23	0.31	0.13	0.10	0.17	0.10	0.22	0.02	0.17	0.06	0.91
262-D2-9/12	37.17	0.25	1.00	0.07	0.05	0.09	0.10	0.45	0.08	0.05	0.08	0.57	0.02	0.97	0.01	0.00	0.01	0.23	1.48
264-D2-9/19	17.23	0.69	2.77	0.03	0.02	0.38	0.16	0.69	0.09	0.22	0.10	0.67	0.01	1.63	0.02	0.00	0.04	0.43	3.34
210-RF-6/9	3.07	2.23	1.32	0.03	0.03	0.26	0.06	0.32	0.18	3.11	0.01	0.04	0.00	0.94	0.15	0.00	0.02	0.11	1.72

[a] The "Other" column represents the sum percent of all remaining identified Orders.
