# Supplementary material for: Correction: Longitudinal Metagenomic Analysis of Hospital Air Identifies Clinically Relevant Microbes
Source: PLoS One. 2016 Dec 28;11(12):e0169376. doi: 10.1371/journal.pone.0169376 (PMC5193377; doi:10.1371/journal.pone.0169376)
Supplement: S1 File — (PDF) [file pone.0169376.s001.pdf]

King et al. supplemental – ZovaSeq method

***This document pertains to the version of ZovaSeq used for King et al. 2016**[1] which is currently available for use from Zova Systems (contact robert.yamamoto@zovasystems.com). ZovaSeq has since been updated and further optimized.*

Although ZovaSeq has several features, the only one used in King et al. 2016 is the ability to find Microbial ID reads. Though specific parameters can be selected, Microbial ID Reads here were defined as any NGS sequence read that contained a 100% match across a 32 nucleotide stretch to only a single taxon within the NCBI taxonomy database and differing from any other taxon match by at least 2 mismatches.

ZovaSeq Method:

Microbial Identification by ZovaSeq takes place by matching NGS reads with taxonomy specific k-mer signatures. Briefly, these signatures can be generated as follows (Figure 1). All possible k-mer sequences are generated from NCBI NT and RefSeq Microbial (Release #48) databases (downloaded on 7/11/2011). All k-mers were then aligned back to RefSeq Microbial and NT databases allowing for 2 mismatches across the entire signature sequence. Alignments were evaluated to determine the most specific taxon identified for each k-mer. Briefly, the most specific taxon identified is either a single taxon to which a unique k-mer signature sequence aligns or, for k-mer signature sequences that align to more than one taxon, the closest common parental taxon. Karimi and Hajdu describes in detail a very similar approach[2] for using HTSFinder to generate 18 nt signatures. ZovaSeq typically uses signatures 30 nt and greater. An example set of 500 *Stenotrophomonas maltophila* signatures is shown in Appendix 1 below.

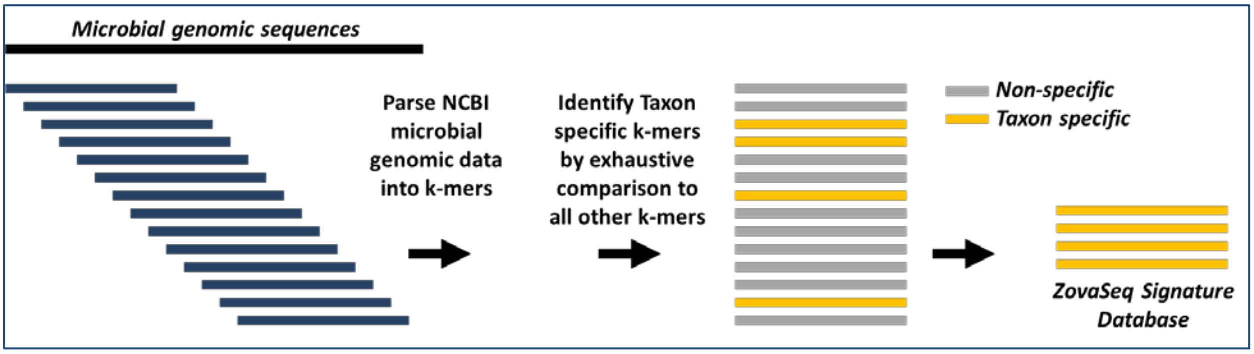

Figure 1. Diagram of Signature Generation steps. Taxon specific k-mers were identified by exhaustive comparison to NT and RefSeq Microbial databases.

After automated barcode and adaptor removal in BaseSpace, Illumina HiSeq generated fastq files (typically 50 nt reads) were directly analyzed by the ZovaSeq platform to identify Microbial ID Reads. ZovaSeq performs an extremely fast search for identical matches between the ZovaSeq taxonomy specific signatures and the NGS reads. Identical results can be obtained using standard string matching algorithms (e.g. *"index()"* in the Python programming language). A subset of Microbial ID Reads for *S. maltophila* is shown in Appendix 2 below. Microbial ID Reads for each taxa are tallied and reported.

[1] King P, Pham LK, Waltz S, Sphar D, Yamamoto RT, Conrad D, Taplitz R, Torriani F, Forsyth A. Longitudinal metagenomic analysis of hospital air identifies clinically relevant microbes. 2016. PLOS ONE;11(8): e0160124. doi:10.1371/journal.pone.0160124.

[2] Karimi and Hajdu. HTSFinder: Powerful Pipeline of DNA Signature Discovery by Parallel and Distributed Computing. Evolutionary Bioinformatics 2016:12 73–85

Appendix 1: ZovaSeq sample signature dataset for *Stenotrophomonas maltophila* (NCBI Taxonomy 40324):

Random subset of 500 signatures. Columns from left to right: NCBI Taxonomy ID, Signature, Index.

40324	AAACGACTGGTGACCCGATAGTAGCCACACTT	232349
40324	AAAGCCTGTACTACAAGACCGAAGGGCACGGC	357038
40324	AACCACGCGTGGCGTGGACCGCTGGAAATCCA	812172
40324	AACCGGCCTTGTTGAACGGAATCGTGACCGAG	948915
40324	AACGATTGGCTGCGGCGCGTCGAGGGCCGGCG	1069852
40324	AACGCCACGCGCCTGTGAGAGGCGCGCGAAAT	1104783
40324	AACGCCGGGCAATGCCCGGCGCTTCGCATCCC	1130539
40324	AACGCGCGCGGCGCAACATCGAAGACGGGGCG	1162024
40324	AACGGTCGGCACCCACCAACAGCAGCTGGCAC	1271551
40324	AACTGGTCGTAGCGCACCAGTTCGCCACGGGT	1447573
40324	AAGCAGCAGGGCATGGACCCGCACGCTGCGGG	1695911
40324	AAGCCCCCGTGACCGAATCGACCGCCACTGAA	1750309
40324	AAGCCGGCCACGTCGGCAAGGGCATCGAGCAG	1789273
40324	AAGCGGCCACCTTCTCCAGAATCCGCTTGCCG	1888135
40324	AAGCTGTTCTACCCGGCATACAACGACTGGAC	1970670
40324	AAGGCCGACCGCGAGCTGCGCATGAAGCAGCA	2058951
40324	AAGGGATCCGACCGCAAGTGCTGGATTACGCC	2136092
40324	AAGGGCCTGGACTACATGCATTTCCAGGCCGG	2146740
40324	AAGGTCAGGGCGATGGCGGGCAGGGCGGCCAG	2183476
40324	AAGGTCCAGCCCAGGCCCACGGTGCTGTCCAC	2185430
40324	AAGTCCCAGCACTGGCGGATGCGCAATGCGAA	2264260
40324	AATAACGCGTAGCGTTACTGCTTGAGCCGGTC	2373483
40324	AATAGAGCAGGAAGCGCTTTTCGCCCCAGGTC	2420741
40324	AATCGCCCGTTCTGGGTGATCGGCACGGTCTA	2569909
40324	AATCGGGCTGCTCGCTCCCAGGTTGCAGGGCC	2601497
40324	AATGATCTGACCGGACGCTGGGACACCCGTTG	2672590
40324	AATGCATCGCTGAGCGCGCTGCTGCTGGCGTC	2691584
40324	AATGGACATGGAAATCTGTCTGTGCCGGCCCC	2762823
40324	AATTCGAAGTCGGCCTTCTTTCCTACCGACAG	2872499
40324	ACAACAGGAAGCGATGCAGGTCCAGCGCGTGC	2996562
40324	ACAGATAGGTCACCTGCCCCTGCAGGTCCTTC	3377477
40324	ACAGCCAAGAACGAGCCGCGACCGCTGCGGGC	3422524
40324	ACAGCGGCGGCAGCGGCAGCGGATCGACCTTG	3476443
40324	ACAGGCGCAGCGGCAGGCGCATGCTGACGATC	3535080
40324	ACAGGGCTTCCAGCAGCGCGCGGTCGCGGCTG	3552647
40324	ACAGGTCGGCGAAGGCGGCATCGACCGCCGCG	3563726
40324	ACATCACCCGGCGCGCCCCCATCCGGTCCGAC	3622535
40324	ACATGGGGAGTGACGGCCAGTACACGCTGAGC	3746421
40324	ACCACGCCGCCGATCACCGCCCCCCAACTTTC	3925153
40324	ACCACTGCCGCAGCTGCGGGCCTACACCGTGG	3958331
40324	ACCATCAATTTCATGTTGCTTCCTTGTTGTAC	4106468
40324	ACCATCCCGACCTTCGAGAAGGAGAGCGGCAT	4114628
40324	ACCATTCGCCCTGGATCTGCTTGCGCACGCGG	4157446
40324	ACCCGCTTCAGCGAGCAATGGGGCTTCGGTAC	4332302
40324	ACCCGGCAGGTTGGTCTGGCCGCGGTCGTCGA	4342154
40324	ACCGAAACCTGCAGCGGCGAACCGATGAACTG	4424069
40324	ACCGACGCGGAGAAGTCCAGGCGTTCGCTGTG	4468469
40324	ACCGCAACGCTCTGGCGCTGCTGGCTGGTGGC	4531520
40324	ACCGCGCCCGCGCCGCCGGTGCGGTGGTCAGC	4660720
40324	ACCGCGCGTTCAATCTGGCGCGAATCGAGCAC	4673882
40324	ACCGCGGGCGACTGATCGACCACATCCAGCTG	4687390
40324	ACCGCTGGTCGGCTGCTTTTGCTCTCGCCTTT	4721646
40324	ACCGTCCGTGTTGCCGGACGCCGCTGTGGTTG	4896118
40324	ACCTGCGGCACCTCCTGGCCGGGCAAGGGGAA	5081186
40324	ACCTGCTGGATCTGCTGCCGGGCGAAATCCGC	5094453
40324	ACCTTCGCCGAGGAAGGCGACGAACGCCAGCA	5172882
40324	ACGAACTGGCCGCGACATCGCGCTACAAGTCC	5244434
40324	ACGCCTTCGGCGAAGCGCGCAGCCTTGAACTC	5791847
40324	ACGGCAGGCGCAGCGGCGACGGTTTCGGTTTC	6135761
40324	ACGGCCGCATCGCTGAGCCCGAGCAGGTCGGC	6170970
40324	ACGGTTACGACGACCAGGGCGCACGTTTCGGG	6389837
40324	ACGTCAAGAGCTGGCATTCCGGGGGATGGCGG	6432988
40324	ACTGCAGTCACCCGGCTGGGCCTGGCCACGCT	6875350
40324	ACTGTTCCTCAGGCATTTCCCGGCGCTGGTCG	7077430
40324	AGACAATGAGGGTCTGCAGCAGCGCAACGACG	7387846
40324	AGAGGACACCTGCAAGGTGCGCGTGCATTCGC	7612691
40324	AGATGGCCCAGTGCCCAGAACGCGCACAGCAT	7807685
40324	AGCACGACGCTGACGTAGACCGGCACCACGGC	8008853
40324	AGCAGGCGCACTTGTCCGCCGGGGATTTCGTC	8177024
40324	AGCCACTTCGGCTTCGTGGGCATGCGGCAGGC	8336931
40324	AGCCCAGCGACAGGCCGCCCGCGGCCGGGTCG	8400376
40324	AGCCGCTGGCTGGCTTTGGCGGCCAACAGAAG	8551889
40324	AGCCTGACCGAACTGATCGGGGGCCAGCTGGA	8642035
40324	AGCGAACACGTCCTGTCCGCGATGGGCGTGCC	8686284
40324	AGCGCCTGTACGAGGAACGCATCCATCACCGC	8912604
40324	AGCGGCGGCGGCAACGGTGCATCGAACCACGG	9120463
40324	AGCGGTGAGGGTACCGGTTCGATGGCCGGGGT	9179316
40324	AGCTCCTGCAGCGCGACCTTGATGCCTGTGCG	9339539
40324	AGCTGCGCGCCACTGGCAGCCGCCAGCGATTC	9428010
40324	AGCTGCGGTGGCGGGCAACGGCCGCGAACGCG	9435058
40324	AGCTGTGGCGAAGGCGCGATCTGCAGCCGCTC	9510121
40324	AGCTGTTTGCGGTGAAGGACCACGCCTGGCGC	9517209
40324	AGGAGACGTCGTCGCTGCGCTTGTCACGGTTG	9687041
40324	AGGAGGGGCCCTTGACCAGGCCGATCAGCAAC	9710532
40324	AGGATCGCGAAGGTGCCCAGCCCACGCGCGGC	9733321
40324	AGGATTGAGGCAGGTTCATACGCTGTCGCTTC	9766759
40324	AGGATTGATCCACGCCATGCGTGGATGCGCTC	9766839
40324	AGGCACCGCACTGTTTCAGCAGGGCGACCGCC	9794313
40324	AGGCACTGGAGCATGACGACGACGCCGACAGC	9806706
40324	AGGCCAGCGCATACAGCGCCGTGGCGAGTACC	9874515
40324	AGGCCGCCTGCGTGCGAAGACGCCGGTGAAGG	9943986
40324	AGGCCGTGGTCACGCTCGGCCAAGTGCGCGGC	9980770
40324	AGGCTCACATTGCCCACATAGGTGTGCTTGTC	10185090
40324	AGGTGCAGGGCATCATCGGCGCGGCGGTCACC	10570489
40324	AGGTGCCGGGCGTGCTGCATGCCACCTGGGAA	10579976
40324	AGGTGGCGTTGACGCCGTCGCGGATCGGTTGT	10619715
40324	AGGTGGTGGCGCTGATGCCTTCGGTGGTGAAC	10631953
40324	AGTCCAAGCTGCGCGGTGTCGAGTTCGCGGTG	10805832
40324	AGTCCAGTGCCGCCACCGGCGTGGCCGACCTG	10813508
40324	ATACTGGATGGCATCTACACGCCCGTGCAGGA	11450388
40324	ATATAGAAGAACGGGGTGTCCAGTTGCTGCTT	11549993
40324	ATCACCGCAGTGGGGTGGCATTGCAGGCGTGG	11729572
40324	ATCACCGCCGATACATCGCCTGCATCCTGTGC	11731593
40324	ATCATCACACCGACGATGCGCTGTTCACCGTC	11882548
40324	ATCCCGCGCTGGGCATCCCCAGCGTCCACATC	12056152
40324	ATCCGGAACTGATGCCGACCACGCAGCTGTTC	12122576
40324	ATCCTGCATCACGAAACCGGCCTTCTCCACCG	12185374
40324	ATCGAAGAGAATCGTGGCGAGCAGGGCATGTG	12233096
40324	ATCGACGCGGAACACGCTCTCCACGGCAGTGC	12277275
40324	ATCGAGGACTACAAGCGCAGCTTCGCGACGGT	12307697
40324	ATCGTACTCATGGTTCCACGACGTTGGGCAGG	12694740
40324	ATCTACAGCAAGGACACCGTCGAAGCCGGATT	12776448
40324	ATCTCGGCTCCGGCTGGGTTCGTGTCTGCATC	12829166
40324	ATGAAAAAGACCCGTTCGTCCAAGCCGCCTGA	12979447
40324	ATGACCAGCCCTCGCGCGCCTTCGCGCTGCTG	13045687
40324	ATGACGTACTCGTAGTCCAGATACTTCAGACC	13081163
40324	ATGACGTGATGATCAGCTTTGCCGCCACCGGA	13082916
40324	ATGAGGAAGACCCCTCACATATTCAACAACGC	13112312
40324	ATGCCCGCGCCCTCCACGCCCGACTTGGTAGC	13312660
40324	ATGCCGGCGAACACGGTGGCCGGGTTGTCCGG	13374758
40324	ATGCGATCCTGTTCGGACAGCGCCAGGTTCTG	13431017
40324	ATGCGCGTGGCCTGGGCTGTCGTGCAGCCACC	13493320
40324	ATGGCCGCTGGCGCGTGGACGCCAAGGACTAC	13756850
40324	ATGGCGATGAGCCCAAGGAAGCGATGGTCGGC	13784084
40324	ATGTAGCCGCCCTCCTCCAGCAATCGCACCCG	13982899
40324	ATGTCACGCTGGGCGCAGGCGCTGGCTTCGAT	13996732
40324	ATGTCGCCCAGGGCGGGCGCGGCCTCGCCAAG	14028604
40324	ATGTGCAGCGGCGAGGACATCGAGGCGCTGGT	14066292
40324	ATGTGGTCGGCATTCCGCCTTCGGCGCGTCCC	14101003
40324	ATTACCTGGTGAAGCAGGGCATCACCACCGAG	14175533
40324	ATTCCAATGTCCGCGCAGGACAAGGGATGTCC	14245715
40324	ATTGCCGAAATGGTGGCCGATGCAATCAACGC	14458473
40324	CAACAGGCCCGGCAGTACGAAGCCCAGCAGAT	14965423
40324	CAACCAGTACTTCGGGCCGGGCAATGGCCCGT	15010859
40324	CAACGACCGTTATCTCGACCCGGTCGACGGCA	15100648
40324	CAACGGAACGCGCCGGCAACCCAGTGCCGGCG	15181516
40324	CAAGGCCACCGAGAAGGGCGACGCACCGGCCA	15515097
40324	CAATCGACACCACCAGCGGTCGGCTCGCCTGT	15706939
40324	CAATGCGTTGCACATCTGGCCGCGTGGCCACT	15793810
40324	CAATTGGCCTGGGAAGTCTGGTTTCTGATTTT	15862879
40324	CACAGGTTGGCGATCATCCGCACTTCGCGCTG	16027513
40324	CACCAGTTCCGGCGTGCGTGGCAGCAGCGCCA	16232285
40324	CACCGATGATCGGATGGCGCACATGCGCCATG	16419929
40324	CACCGCCACCGGCAACGGCGCGTGGCCCACGG	16448924
40324	CACCGCGTAGTCGATCTGGCCAAAGCCATGTG	16506288
40324	CACCGGAGACCACGATGTTCTTGCGGTACATC	16525934
40324	CACCTACCCGCCTGCCAATGCAGACCGCATCG	16636235
40324	CACCTGCACCTGCAGGTCACCGCCTATGGCGA	16671657
40324	CACGCAGTGGCCGCTACCTCAGCGCCATCGTG	16852007
40324	CACGGTACCCACGCCCAGGCCGACGCAGACCG	17126477
40324	CACGGTATGATGCCGCAACCCCGGCACTTGCA	17128506
40324	CACGGTCGGCAGCTCACCCAGCGCCTGTCCCA	17135448
40324	CACGTGCGAGCCGATGGCGTAGTCCGGTGTGA	17206161
40324	CACTGGAATCCAGGCGCCTGGACTTCGATGAC	17357568
40324	CACTTTGGCCAGCTGTGGCAGAGCCAGCTTCC	17437535
40324	CAGAAAGTCACTCACGCATGGAGGCGAATTAC	17450280
40324	CAGAGCCTTGAAGCGCCGGGCGCTGCGAAAAA	17574854
40324	CAGCAAACTCCCATCGCTTTCCACGTCGGGTG	17691960
40324	CAGCATGCCCCTCCTCGGCGGTCCATGCGCGC	17934587
40324	CAGCCCTTGTGCCTGCAGCGGCGGCAAGGCGG	18037640
40324	CAGCCGTCGCCGCAGTGGCGCCTGACCGCCGG	18098519
40324	CAGCCTTGCTGCGCTGGCGCTGGCGGATCAGG	18130614
40324	CAGCGCGGTTTCGGCCGGGACCAGGAACGACT	18308276
40324	CAGCGGCCCCACGCCGATGATCGCCGGCTGCC	18358557
40324	CAGCGGCCGGCACTACCGCATGCCACGCCGTA	18361640
40324	CAGCGTCGATGTGGTGGTAATCGGAGGTGGCC	18424495
40324	CAGCTGGCGCTGGACTACATCACCTTCGGCAT	18559362
40324	CAGCTGTGCCCACAGGTCGTACAGCGCGATCT	18573116
40324	CAGGAAGCCTCGCGGTCCGTACATGCGGTTCC	18626976
40324	CAGGCCACCGCGCCACTGCTGCAACCGTTCGT	18747177
40324	CAGGCCGCTGCTGAGCTTCACCGTACCGAGCT	18798805
40324	CAGGCCTGGCGGCACCCGCGCCCGTTGCACAC	18821674
40324	CAGGCGCAGGCAGGGCGAAGCGAGCCTGCTGG	18846154
40324	CAGGCGCTCGGTCAGTGCTTCCTCGCGCTGCA	18870356
40324	CAGGGTGTAGCCGCCGCCCATGCGGTTGCAGG	19036113
40324	CAGGTGACCGTCTGGCCGGCGGTCAGTGCCGG	19105792
40324	CAGGTTTCGTTCCACGGTGATGCGCGGCCCGA	19167924
40324	CAGTCCTTACAGGGTTGACCGCCGATTATAGC	19230317
40324	CAGTGCACCAGCCTTGCGGCCTTGTGGCGCAA	19277821
40324	CAGTGGCCAGGAGTACGCGACACGTCATGGTC	19328418
40324	CAGTGTCTGCGGCAGGCGCAGCGTGAAGCGCA	19353626
40324	CAGTGTGCAGCACGGCACGCAGTATTCAATGA	19354615
40324	CAGTTCGTCGTTGCCACGCCCCGGCGGTGGCG	19393248
40324	CATCACCACTGACCAGCCCGCTGCTGGCCATG	19556988
40324	CATCGCCACCTTCGGCAGTGTCGCTGCAGCCG	19817805
40324	CATCGCGGTCCGGTGCCTCGTAGCTGAGCGAA	19872092
40324	CATCGGCACCTTGAACACGCCGAACTGGGCAC	19896552
40324	CATCGGTGATTACTCCACGGCATTGGGTACGC	19943677
40324	CATCGTCGTAGGCGCGGAACACCACATCCAGC	19964672
40324	CATGAACGGGTTGCAGGCCACGGCCCGCTTGA	20054603
40324	CATGCGATGCGTGGTAGCCGATGGTCATCACG	20189520
40324	CATGCGCTCGAGGATGCCGAGCAGGGAGTTCA	20210868
40324	CATGCTGGGCTTCAGCCTGATGCTGGCGGCGT	20250428
40324	CATGGCAACGCCAATGGCAACCACCGCGACCT	20276089
40324	CATGGGCGATGCGATAGCCGATCCAGTAGCCG	20328437
40324	CATGTCGATCACTGGCCTGGTGGCCGTGCTGA	20378522
40324	CATTCGCTGGCCGCCGAAGCATCGAGGATCTG	20466235
40324	CATTGGCCGCGCTGGGCCTGCCCTTGGCCGGG	20547762
40324	CATTGGGGAAGAACAGCTTGATGTGGTCGTCA	20557800
40324	CCAAAGAACGCCAACACTGCGGCCAATGTCGC	20624829
40324	CCAACACGGTGACTCTGTCGCTGGCCAACCCG	20652879
40324	CCACACGCACCTTGGCAGGATCGATGCCGAGG	20923076
40324	CCACATGGCGTACTGCCAGGCCGTCGGCCAGG	20966605
40324	CCACCCGGATGCAGCACGGCGAGGCGCTCGAC	21048497
40324	CCACCGCCTTGTCGACGCCCGAAGATGCCAAC	21091074
40324	CCACCGGCTTGCCGTTCTTCACTGCGGGCCGA	21117986
40324	CCACGCATAGCCCTGCCAGCTGATCGGGCCCA	21200959
40324	CCACGCCCACCGGTGCGCCGATCCACTTGTGC	21209712
40324	CCACGTGCCGGGCAACCATGACCTCGATTTCG	21317452
40324	CCACTGTTGCCGCACGGCTTACAGTAGCGTGC	21373864
40324	CCAGCACCGTGGCGGCCAGCGCCCATGGCAGG	21505169
40324	CCAGCATCAGGTCCGCCTGCGGATCACGCAGG	21556710
40324	CCAGCCGAAGGCCGCATTCGGCGACCAGCCGG	21600680
40324	CCAGCGGCACGCCCATGTAGCGGCTGTGCACG	21710578
40324	CCAGCTGCCGAAGATTCCGATGGGCAACAACG	21773605
40324	CCAGCTGGCGCGTAGCCCAGGCCAACTCGCTG	21783329
40324	CCAGGCGCGAGGCCTCCAGCTGCGGCGGCAAG	21881329
40324	CCATCGCCGCACTGGCCACGCTGGAGGATGCG	22178728
40324	CCATCGTCGTAGGCGTTGACCGGGACCAGCCA	22212935
40324	CCATTCCACGATGCGCGCGATCACGCGCAGGG	22346545
40324	CCCACGTTGCCATCCACGACGTGCGCCAACGC	22511949
40324	CCCAGCCGGCCAGCGAAGTACGTTTCATTGCG	22564214
40324	CCCAGCTGCCGATCAGCGGATAACCGCCCTCG	22591768
40324	CCCCAGTTCGGTACAACATCAACTTTCCCCGG	22748321
40324	CCCCCATGAAAATCCTGTACACCAACTTCCAC	22768828
40324	CCCGCCCAGCGTGCCCGGCGGCCTGGCCTCAC	23019294
40324	CCCGCCGGCACCGTTGCGGCGATACCCTGCAG	23036574
40324	CCCGGCGCGCCTTCACTTCGTCGCCGATCCGC	23183275
40324	CCCGGCGGGGTTCTTTAGAGGAATCGACGCGG	23190230
40324	CCCGTCGTGATGGCGTACGCCTGGAAGTGGAC	23270690
40324	CCCTCCAAGGACGACCCAACAGGGAACATGCA	23323537
40324	CCCTGGGCCTGCGCCTGCAGGGTCTCGGCCAG	23431074
40324	CCGAAACGCCCAGTACATCGGCGGGCAGCAGG	23501065
40324	CCGAAGACAAGGCGCGCATCAGTGCCGAGTAC	23546548
40324	CCGAAGTCACCCATTTCCCAACCCAGGTTCAG	23577339
40324	CCGAAGTCGGCGGCGGCGCCCACCACCAGGCG	23578719
40324	CCGAATCGCGGCCGCTGGACCCTGCGCAGGAA	23589960
40324	CCGACAGCTACAGCGCGATCGGCGAGCGACCG	23622633
40324	CCGACGCTCAGCTGGCCGCACCTGATCGCGTT	23709159
40324	CCGAGATCGGCGAGCAGGACCAGTCGCTGCCG	23758572
40324	CCGAGCAGGGCGGTGGCTGCCTCGTTGAACCA	23772666
40324	CCGATGACCAACACAGTCCTGCGCACACTCGC	23921272
40324	CCGATGGCCTGTGGCAGGACGTGCTGGCGCAG	23960284
40324	CCGCACGCACCACGCCATCGAGCACTACATCC	24061869
40324	CCGCAGGTGGCCCGCAAGCTGGGCATCGGTAC	24130676
40324	CCGCAGTTTCTCCGACGTGCCGATCCTGATGC	24139954
40324	CCGCCGCGCAGCTTGTCGATGGCCGCCTGCAG	24352335
40324	CCGCCGCTGGCGTTCCCGGAAGAAGCGCAGAA	24366409
40324	CCGCCGGCATCCGCGCGGCAGTGCTCAAGCGC	24375656
40324	CCGCGAACGCTACGACGTGGTCAACACCACCA	24457727
40324	CCGCGCCCGGTCGAATTCCTGCCGGCGATGCG	24544988
40324	CCGCGCTGGCAGATGCGGAGTCCGCGTTGGCC	24616785
40324	CCGCGGTTTCCCAGCATGCCCGCCGCAACGAA	24675385
40324	CCGCGTGGATGCCGATGAAGTGGCCGACGCGA	24703298
40324	CCGCTGCGCTCCAGTGCCGCGCTGGAATCCGT	24792138
40324	CCGCTGGCCATGCTCTGCCATGGGCTGATTGG	24810694
40324	CCGGAGGGCTTTGGCGACCTGCATGGCAAGTT	24934976
40324	CCGGCACCGAGGCCGCCGATGTGGCCGACCAG	25000438
40324	CCGGCAGACGGCCGAGCATCGGCCGTTGTGAC	25020506
40324	CCGGCCACTTCCAGCAGCGGCTTGGGCGTGTG	25080089
40324	CCGGCCCGGAATCCGGGAAGCCGCCGCAGGCG	25106850
40324	CCGGCCGATGCCCACGAAGGCTTCAAGGAAGC	25117188
40324	CCGGCGACGATCTGGTTGACCAGCGCGCCACG	25172623
40324	CCGGGAACGGGATCGCGTCGGGGCTGGCGTAG	25337670
40324	CCGGTGACAACGGGCGCTCCGCCGGGGCGACG	25523195
40324	CCGGTGTGGAAGCGTTGACGGTGCATTGAGGA	25590134
40324	CCGGTTGCCGGCCATCCCGGCCAACTGCAGGT	25605736
40324	CCGTATGTGGGCGGTCGGCTGTCGACCATCGA	25658462
40324	CCGTCGAAGCCGGCCTCCTGCAGCAGCGCCAC	25704640
40324	CCGTCTTCGCTCAGCGCGGTCAGCGTGGCGCT	25756341
40324	CCGTGCCGGACCCGCCGGGTGGCGAGTCGGTG	25792805
40324	CCGTGCTGCGCCGGCTGGTGGAGATGAAGCTG	25813890
40324	CCGTTTCCTCCGGGAAACGGATCTGACCCCTT	25936687
40324	CCTCGCGGTGATCGCCCGCACCGAGCGCCTGT	26165823
40324	CCTCGGCCTCGTCGGTAAGGATCACCGCCGTG	26186007
40324	CCTCGGTCACTTTCGATCCTGATGGCAGCGGC	26199528
40324	CCTGCGAGCGCGGCCGCGCGCGGACCGCGTGC	26435034
40324	CCTGCGATCTGTCGGGACGGCGTTCTGCGTTG	26436593
40324	CCTGCTGCCGCTGCTGGAGACGCAGCCGGAAG	26530128
40324	CCTGGACTGCACCGAGCGGCGCCCACACCTGG	26580867
40324	CCTGGCGGACATTGGGATCCTTCTCGACGGCC	26649287
40324	CCTGGGCAACTTAGAGTGTGGTAGAGGGTAGT	26671941
40324	CCTGGTCCCAGTGCGTGATGCGCTCCAGCAGG	26713537
40324	CCTGTTCGCCGAGCGCCGCGCGGCGATGGCTG	26793158
40324	CCTTCGATGGCCGCAAGGGCCGTATTTCGCAG	26872315
40324	CCTTCGCGTGTCGGTGGATACGGCAGTGGCGA	26882116
40324	CCTTTCCCGGCAGACCGTGGTCGGCCTGGAAG	27037309
40324	CCTTTGCTTCTGCTCACCCCTCCGCCGCGTGG	27047818
40324	CGAAACGCGCCTGCCCGAACTGGCCTGCTCAT	27087411
40324	CGAAATCACTGGCGGCGGTGATGGCGGCATCG	27108241
40324	CGAACGCCCCGACAGCTGCGACTGCAGGCCGC	27191634
40324	CGAACTGGATTGTCATGCACGCTGAAGTCCCC	27234350
40324	CGAAGATGCCTCGACAGGCCCGGCCCATGCCG	27273325
40324	CGAAGCAATCCATACTAGCAAGTATGGTTACG	27276624
40324	CGAAGCCAAGCCGCACATGCCCGAATGGGGCG	27281752
40324	CGAAGCCGGCCAGTCCGGCGAAGCAGTCAAGG	27293795
40324	CGAAGGCAAGCAGGGCGAACAGCGCGGCCAGC	27327158
40324	CGAAGGCGCGCGCGGTTGCCTGCTGCGTGTGC	27340874
40324	CGACATCACGCCGCGCTGGTAGGCCGGCCGCT	27540805
40324	CGACCCACGGCCGCGCCGCTTCCCAGAACTTG	27621416
40324	CGACCCGCAAGCCGGGCATCGGCCTGATGACC	27632132
40324	CGACCTTTACTGGAACAGCAATGATGGGTTGC	27730946
40324	CGACGAGCGGCGCGGTGGGATCGGCGGGTGGG	27762407
40324	CGACGGCGCTGCGCGTGCTGCAGCAGTTCCAG	27859861
40324	CGACGTGGTCGGCGAGGTGCATGGCTTCCTGG	27902404
40324	CGACTGAGCGCACTGGGCGTGCTGTTCACCGG	27932631
40324	CGAGCCTGCTGTTCGCTGCGGCCCACGCCGCC	28103979
40324	CGAGGACGACAACGACGCCGACCGCGCGGTGG	28191834
40324	CGAGGATCGCGCCCAGCACCATGCCGGTGGGC	28200686
40324	CGAGGATGCGCAGGTGGCTGGACAACGCCGGC	28202349
40324	CGATCGGCTCCAGCAAGGCCTGCAGCAGCGGC	28526876
40324	CGATCTCACGCAGCACCGCGTCCATCGGCCGC	28543949
40324	CGATGAACCCAGTGCTTCCTGCCAGCCCAGCG	28584928
40324	CGATGCACCTGATCTACCTGCCGGATCGACGG	28626855
40324	CGATGCCGCCGCCCCGGCCACCACCGAGCAGG	28656191
40324	CGATGGGCATAGCGGCCCACCATGCTGCCAAA	28762729
40324	CGCACGCAGGCGGCCATGCAGGCTGGCCCAGT	29132952
40324	CGCACGCTGCCGCCCGCACTGGACGTGCGGGT	29156669
40324	CGCACTGCTGGAGCCGGCGCGCACCTTGCTGG	29197872
40324	CGCACTGGTGCTGCAGGAAGCCTCGCGGTCCG	29203599
40324	CGCACTTCACCGATGCGGGCAGAGGGTTCCAT	29205914
40324	CGCAGCCCGCTGGCGGTGCAGGTCGGGTTGGC	29280455
40324	CGCAGCTTCGAGGCGGGCCACTGGTCGCAGGC	29348946
40324	CGCAGGACGTGCCGGAAGAGGAAGAATAAAGC	29357997
40324	CGCAGGGAGCATGCTGCAGGCGGCCGTCTTCG	29397960
40324	CGCATCCGCGACGCCGTTGAAGCGGCGCTGTC	29497279
40324	CGCCACTTGCGGTGGCGGCGACTGCCCACTAG	29747953
40324	CGCCAGCAGCCCGGCGATGCGCTCCGCTACCT	29766730
40324	CGCCAGCAGCGCCTGCACCTGGCGGCCGGCGG	29767289
40324	CGCCAGCGGTTCATCCAGCGCCTGCCGTGCGC	29797688
40324	CGCCAGCTGCGGCGCCAGCAGGCGGGCCTGCG	29804588
40324	CGCCATGCGATTCCCCGCTGGTACGCGGACGC	29879783
40324	CGCCATGGCGCTTACCAGCGCGGGGTCTTGGT	29885239
40324	CGCCCAGGCATCGCGATCGGACTGGAACAGCG	29938586
40324	CGCCCGCCTGTATGGCATGACCCCGCACGGCA	30007904
40324	CGCCCTGGCCAGCAAGCCGCGCCTGCTGGTGA	30069664
40324	CGCCGAAGCATCGCCCAACGCCACGACGCTGC	30095662
40324	CGCCGAGCTGGAAGGGCGCATTCCGCCGTGGT	30145024
40324	CGCCGCCCCCCCGGTGGCGTCGCGATGGACCG	30227605
40324	CGCCGGATAGTGGCGGGCCAGGAAGCCGCGCA	30348051
40324	CGCCGGCAATTACCCGTCGCCGTATGGCGATG	30355885
40324	CGCCTGCGCGCACCGCAGCAGGCGGCAATCAA	30596206
40324	CGCCTGCTGCGCGTCCTCCCAGCCACGGCGCA	30608244
40324	CGCCTTCATTGATCCAGCCCACTGCTGCAACA	30652098
40324	CGCCTTCGGCGCACCGTCCACCAGGTACTGGT	30662144
40324	CGCGAACAGGGCCGCCAGGAAGTGATCGTGAA	30695737
40324	CGCGAATCTACTTCGTCCAGCTGCAGCTCAAC	30719851
40324	CGCGATGGCCGCCTGCAGGTGGAAAGCAGCGC	30859451
40324	CGCGCAGATCGCCAATGGCGCCATCGAGCAGA	30930529
40324	CGCGCATCGGTGACACCTTCTGGAAGCGCTAC	30981714
40324	CGCGCCAGCACGTACTGGGCCACATTGCTGCG	31010634
40324	CGCGGCAATCCATGCCTCGGTGGTCATCGTGT	31394510
40324	CGCGGGAACGATGTCCTGCGTGGGATGCGGTG	31519398
40324	CGCGTACAGGGCCGCTTGGCCCCGGCCGAACT	31627890
40324	CGCGTCGCGCACGGCGGTCACGCCCGGCACGC	31671152
40324	CGCGTCGTCAGCATAGCCATACCTGCGGATGG	31679569
40324	CGCGTGCCCAGTGCGAGCGTGCCGACCAGCAG	31703297
40324	CGCTGCGCTGAACCGTGGCAACAGCCGCCTGG	32093405
40324	CGCTGGCCTGAAGGCCAAGGGCAAAGCGAGCA	32186124
40324	CGCTGTCGATGATTTGCAGCATGGCCTGCGCA	32267193
40324	CGGACCGGGTGCCATTCCTGGTGCTGGCACTG	32508217
40324	CGGAGAACGGGAACGGGAAGCGGCCTTCCAGG	32553241
40324	CGGAGACCGCGTTGTAGTTCCACGTGAGGTTG	32556685
40324	CGGATCACGAAACTGTCACCGGCAGCGAGTTG	32619625
40324	CGGATCGTTGTCCAGATTGGCGATCACCACGC	32643813
40324	CGGATTTCTTCCTCTTCCGGTGCGGCCTGCGG	32695790
40324	CGGCAAGGACCAGCAACCCGATGCCCAACCTC	32744786
40324	CGGCACGCCACCAGCCTGTTCCACCATTTCCA	32819283
40324	CGGCACGCGGCAGGGCATAACTGATGGCGACG	32825281
40324	CGGCACTGCCGGTATCGTCGTCATCGTCGAAG	32844045
40324	CGGCAGCGGCACGGCGCACCTGGCGGCGCTCG	32888646
40324	CGGCATCGCTGCGCCCCATGCGCAGCAGGCCC	32961184
40324	CGGCCAGCAGGGGTAGTTCCGGCCGCTGGCCG	33063164
40324	CGGCCAGCGGCCGGCACTATCACTACGCCGCG	33074659
40324	CGGCCATTGCCGAAGTACACGGCAAGCGCCAG	33116552
40324	CGGCCGCACATGAAACCGTGGCTGAGTGCGGA	33184043
40324	CGGCCGGCAGCAGGCCCAGGCCATCGCGCAGG	33230233
40324	CGGCCGGCCTCGGCGGTGCGCGCGCCAACCTG	33234616
40324	CGGCCGTGTTCAAGGAGGTGATGCAGGCGACG	33265352
40324	CGGCGATTGCCTGCCTGGATGAAGCGCTGGGT	33432806
40324	CGGCGCACGCCACAGGCGGCCCTCAAACTTCG	33443059
40324	CGGCGCAGGCCGCGGGCCTGGCCCAGCTGGCT	33453096
40324	CGGCGGACTGGATCCGTTCGAGGTGGGCCTGT	33584445
40324	CGGCGGCTTCGCACCGATCCAGACCCTCAACC	33649034
40324	CGGCGGGGGCGAGGCGGGGGCGCCGTCCTTGT	33661277
40324	CGGCGTCGATGCCACGGCAGTGGCCGCACTGA	33710563
40324	CGGCTCGCTTGAAGGCGGACCCAACAGTCGCG	33807680
40324	CGGCTGCGGCAAGCAGGGTCAGCCCTGCGCAT	33845435
40324	CGGCTGGACAACCGCGCCGGCACGCTGGGTAC	33854972
40324	CGGCTGGCATGGGCCAGTTCCTCGCGTTGCGG	33860422
40324	CGGCTTGGCGATGGCATCGTGCTGCTTGGCCA	33917755
40324	CGGGAAACGCCCACTATCCCAGAGCCTGCCAG	33929370
40324	CGGGATGCCCAGCAGTGACCATGCCGCCAGCC	33977698
40324	CGGGCAAAGCCATCGAGCTGTTCGCGCAGCTC	33987495
40324	CGGGCCGCCTGCTGCGCTGCCCGGGTGGTGGC	34054809
40324	CGGGCGCGTCAGTTCGTAGACGAACGTCTGGT	34104240
40324	CGGGGCGTAGCTGAGATCGGCCGGCACATCGG	34197324
40324	CGGGGCTGCAGGTCGATGCGATTCTCTCGTAA	34200228
40324	CGGGGGATAGGAGGAATGCGTGGACCTGAATT	34204399
40324	CGGGGGCTGCGATGCCTTGGCGGGCCCAGATC	34209107
40324	CGGGTACTGGCCAAGGACTTCCTGGACCGCGT	34242035
40324	CGGGTCCAGCTTCAGTGCCAGGGCAAGGTTGG	34256603
40324	CGGGTGACCGCACCGGGACTGGCCCGCATCAG	34280095
40324	CGGGTGACCGTTGCACGTGCATTTCGATGGCC	34280304
40324	CGGTACGGATTGTTCCGGGCTGCCGGCCGATT	34361651
40324	CGGTCAGGTAGACACCGGTCGAGCTGTAGTCC	34432098
40324	CGGTCGATCCGGAGGTGGAAGCCGAGCGCATG	34469438
40324	CGGTCGTGGCAGCGACGAAGCCATTGATCTGC	34505549
40324	CGGTCTTCATCAACGGCCTGAATGTCACCGAC	34525632
40324	CGGTGATGCGGCGCACCGGCAGCACCGCCCAT	34579743
40324	CGGTGCGCCCGACCTGCATGGTGGCGTAGGCC	34635228
40324	CGGTGCGTTGCCGCAGGCGCGAGTCGGCCGGC	34656732
40324	CGGTGCTGTCCGAATAGGCCACATCGATGTCG	34671198
40324	CGGTTCCAGCGCATCCAGCCAGGACAGTTCCT	34804040
40324	CGGTTGGTCTGCGCAGCAGCGGCAGCCTTGGC	34844499
40324	CGTAGCTGCCCAGCAGCGCGGTGAAGTCGTAG	34985934
40324	CGTCGCGGCTGCCGAAGCTCTCGTCCTTCGGG	35315782
40324	CGTCGGCGGTGAGGATCTGCAGCAGTGCCACT	35356707
40324	CGTGAAGGACGTTCGAAAGGGGCGACGGGGGA	35470615
40324	CGTGCAGTTCCTTGTTGACCCACTCGACCGTT	35567741
40324	CGTGCCGTGCCTTCACGCCGGCCACGTCCCAG	35621687
40324	CGTGGGCCGCCGGCCTTCATGGCCGCCTGAGC	35842979
40324	CGTTCACTTCGGCGAAGCCCTCGCCACTGACC	36002337
40324	CGTTCATCGCCTCGCTCCAGGCCGCGTCGTTG	36006543
40324	CGTTCCGTGCGCACCACGTGTTTGCGCTGCAG	36024880
40324	CGTTCTGGCCATTGCGCTGCTGTTCCTGCACC	36075472
40324	CGTTGGTCCGCACGACACGGCAGTGCCGGCCG	36200908
40324	CTACGCCTTCGCAGTAGGGCTGGATCGGCACG	36434297
40324	CTATGCGGTGTCGTCGCTGCGCCGACGCATGC	36586445
40324	CTCAACGACACCTTCAAGATCCACCGCGGCAG	36636981
40324	CTCAATGTCGAGCGCGCGCGTGGCCAGTACCG	36673405
40324	CTCCAGCCCCGGTGGCGTGCCCGAGCCGGTGT	36845623
40324	CTCGAACTTGCGTACCCAGCGTTCGTAGACTT	37007449
40324	CTCGACCGGCGCGTGCCGGTGCGCGATGCCGA	37027478
40324	CTCGCCGCAGACCCTGCAGCTGCAGGGCGAGA	37119050
40324	CTCGCGGATCAGCCAATCGATGGGCGACAGGC	37164076
40324	CTCGGACTCTGCATTGGCATCGACCACCAGCG	37206593
40324	CTCGGGCTCGTTGAGGATGCGGAACGCGATCG	37281206
40324	CTCTCGCCGGGCGTCGCTTCGGCATACGCCTG	37389200
40324	CTGACCGGCAGCATCGGCAGCGGCTCCAGCGA	37547444
40324	CTGCACGAGGGGCTGGCCTTCCGGCTGCCAAG	37789671
40324	CTGCACTGGATCGCCGAGGGTTTCACTGCGAT	37810350
40324	CTGCAGATCTATGCGCTGCAGAAACTGGGGCG	37821476
40324	CTGCATCGGCGGAGATTCATCCGATTCGCGGG	37908009
40324	CTGCATGCACGCGCGCTTGAAGTCGCCCGTGC	37914248
40324	CTGCATTGGCATCGACCACCAGCGGCAACGGC	37928136
40324	CTGCCAATGCGGGTGGCTGGGAAGAGTTCTAG	37935759
40324	CTGCCCTACGAAGACCGCCTGCCACTGCGCGA	38000118
40324	CTGCCGGCACGTGGTATGTGCGGGTGAAGGCC	38056874
40324	CTGCGCGCCAGTGGCGATACGATCACCCGCAG	38217683
40324	CTGCGGTAGGAACGAAGCAGCGGCAGCGTGCC	38312657
40324	CTGCTCCAACAGATCGCGGAAATCTGTCGAAG	38375463
40324	CTGCTGGCGCAGACCGTGATGCAGAAATCCCG	38474787
40324	CTGCTGGTGGTCACCGAGAATTATCCGGTGCT	38490907
40324	CTGGAAGAATCGTCGATAGACTTCAGGATCAG	38549895
40324	CTGGATGACGCCCACGACCGCCTCTACGTGGG	38659591
40324	CTGGCCTTCTTCCAGCCGCGGCAGCCCGTAGC	38814838
40324	CTGGCGCTGGCCGTACAGCCAGGCCGACAGGC	38869189
40324	CTGGGCCGCGAGGACTACACCGAAGCGCAGCG	38982467
40324	CTGTAGGCCAGTGTCACGCCATCGGCACCGCC	39228964
40324	CTGTCGCTGCTCCTGCACGGTCATGCCGTCGA	39284663
40324	CTGTCGGGCAGGGCGTTCATGGAGGATGTCTT	39292025
40324	CTGTGCCCACGCATCTGCAAGCGGGACCGCAA	39322115
40324	CTTCAGCGGTATGAACGATGGCCCGGGCTGGG	39541671
40324	CTTCAGGTCCTTGTAGAAGATCGCTGCGGCCA	39548246
40324	CTTCGCCACCGATGCTGGCTTCAACATCTGCA	39701427
40324	CTTCGGCCTGCCAGCACACCATCAGCGCCAGC	39750564
40324	CTTCGGCGCGGTGGTGCTGCTCGCGGCGATCA	39755654
40324	CTTCGGTAGGGGATCACGCACATGGATCGACG	39769148
40324	CTTCTACTCGCGTTATGGCTGCAGCAAGGTTG	39802975
40324	CTTGCAGTGGTGGGTGCCGACCGTTGGTCGGC	39938370
40324	CTTGGCAAAGCGCACGACTTCATCGGCGATCG	40059289
40324	CTTGGCCACGGGCTGGATCGTCCGCTGCGGCT	40068144
40324	CTTGGCGCCCCCTGCGGACGCGACGCAAGCAC	40086642
40324	GAAACCACGGCCGGGGTAGACATCGATGGTGT	40376413
40324	GAAATCGGCCTTGTTGTCGACAAAGTCGCGGA	40487999
40324	GAACACCCGGGCCAGGGTGGCCGCGTCCATGC	40541917
40324	GAACCCACCGACCCGGCGCAGAAGGCGCTGCC	40622863
40324	GAACCTGAACGGATTGCAGCTTTTTGTAGTGT	40655275
40324	GAACCTGCCGGACGTGGGCATCACTCCGCAGT	40656963
40324	GAACGCATGCGCGAAGCATCGGAAGACCTGCC	40684991
40324	GAACTCGAAGCCCATTTCCGCCAGCGCGGGGC	40763860
40324	GAACTTGGTGGTATCCAGCACCAGGGTGCCGT	40815337
40324	GAAGATGGAAGAATTGGTGAAGAACCTGGCCA	40893149
40324	GAAGGATGCGCCACAGCGCGCCAACCAGATCG	41029384
40324	GACACCGCGGCCAGCCACGCCAGGCCACCGAA	41432275
40324	GACAGCCGATCGGCCTGTCCGGCAACAGTGGC	41489316
40324	GACAGGTGCACGTCACGCTCGACCGGCTGGTC	41525853
40324	GACCAATGCGCTGGTGGAAGTGATGGACGCCA	41616412
40324	GACCACACCCTGCTGGCGCAGCCGCTCGGCCT	41619069
40324	GACCATCGAACGCTTCGAGTACCCGCGCAGCG	41701158
40324	GACCCGCACTGCCCGGTATGCGCGCCGGGCGT	41750280
40324	GACCCTCACCGGTACGCCGTCGGCATCGGCAA	41774828
40324	GACCGGCCCGGCGCTGCACGGCGACTACGTGG	41872790
40324	GACCGGCTTCACCGGTTTCCCGCGCCCGCGTA	41883170
40324	GACGAGGTGGCCTTCGGCGAGGCCATCAGTGC	42071656
40324	GACGCACTGCGCGAGATGCTGGGCCTGGCCGA	42117303
40324	GACGCCCTGCCCTACGACGCCGACGGCGTGAA	42150498
40324	GACGGATAGCCCTCATGGCTGATCGACGGCGC	42254198
40324	GAGACTGCGGGCCAGCCGGCGGTGACCGAGCG	42624470

Appendix 2: Subset of Microbial ID Reads identified for *Stenotrophomonas maltophila* (NCBI Taxonomy ID 40324)

Random subset of 500 reads; Columns from left to right: Sequence Read, Read Count, NCBI Taxonomy ID

Microbial ID Reads are from NGS sample 262: https://www.ncbi.nlm.nih.gov/biosample/4007375, BioProject: PRJNA287928, BioSample: SAMN04007375, Experiment: SRX1164734, Run: SRR2183729.

AACTCGCCGCGTCACTTCCTTTCCTGGATGCCCTTCATGCTGCGCTCTGC	1	40324
CTACGCGATGCCCGGCAAGGCGCTCAAGGGGCTCTCGGGGTGGTCGGTAC	2	40324
TCGACCAGCTTGCGCGCATCGCTGGCGCCGAACTGCGCGTTGACCAGGAT	3	40324
ATCGGCCAGCGCGTTGCTGAGTGCGGCCGCCGAGCTGATGCGGGTCAGCC	1	40324
GCTGGCCCGCGAAGGCGGGCTTGGTTCGGCGCTGTGGGCCGGCCCCGAAT	3	40324
GCCAACAGCGTCGCGCTGGGCGCGAACTCCGAAGCGGTGCGTGCCAACAG	2	40324
ACCTGGCGGTCATCGCCGGCCCCGGTGGTCGCCGCAGCCTGCGGCGCACG	3	40324
TCGCTGTTGCGTTCGGAGAAGAAGCGCGACTCGCTGCGCACGCGCATGTC	1	40324
GCCGCCGGTGCTGCTGGAATACGACAAGGACTGGGGATTGAAGTCGGTGT	1	40324
GTACACACCCCCCGACAGAGTTCAAGGCGACAGTCGTTCCGGCCCAGTAG	3	40324
TGCACGAAGGTTTCGGCCTGCGCGATTTCGAGTCGCGCACGGGGCTGCCG	2	40324
GCGACGCACTCCCTTGCGAACCTGCATGCGACGTTTCCTTGTCGTTGAGT	1	40324
CCTGCAGGGCCGGCAGGTTGACGATCACCTGGTTGGCGACGCTGGTGGCG	4	40324
ATCGTGGTCGGTGGCACCAACGGCAAGGGCTCAACCGTGGCTTTCATCGA	4	40324
CGCTCGACACCGACATGCCGGACGATGTGGTGGAGCCATCATTGAACCTG	1	40324
ATTCTGCGCCATGCAGCTGCGATAGCCGGCCTGGCACTGCACGCGGCACT	1	40324
GGACAAGACCATGCACGACTTCCCCGATGCGGCGCAGCTGGTGCAGACCC	2	40324
CAGCCCGGCCACCAGGCCATGCAGGGTCGAGGCCGCGATCAGGCGCCAGC	1	40324
CCATTCGGCGAAACAGGCGCACAGGCGGCGCTGTGCCCACGCATCTGCAA	2	40324
CTCAGCGCGCCAGCCAGGCGCCTACGGCCACGGCCACGGCCAGCCACAGC	3	40324
GTGACAGGCCCACGCGCACGCCGGCGTGCTGGCGGCTGCGGGTCAGCAGG	2	40324
CACTGGACCAGGACGTGGACAAGCTCACTGGCCTGCGCGTCGGCGCCGAC	1	40324
GCAACATGGTCGCGTGGCATGCCGCTGGCGACCGCGCGGGCAATCTGTTC	1	40324
GGTCAGCGGCAGGCGGATCAGCGTGCGGGTGCCAGTACCCAGGCTGCTTT	1	40324
ATCGACTCGGTGCGCAACGCCCTCGAGGTTGGCTACCGTGCGGTCGATAC	1	40324
GCCGTTCGACCAGGCCGCATTGCCGGCCGGTTTCGGTGCGCCGTATGAGC	3	40324
CGCAGTGCCGAGCTGGCCGAGCGCGCCGGTTTCAGCGGCGTGGAGATCCA	3	40324
TGCAACAAGGAACGCTCATACCCGGTCTATCGCGAGAATCCTGCACCCAA	3	40324
AACGAGCACGGCGTGGGCGTGCGCAATGCCGGCAGCATCGGCGCGCAGGC	1	40324
CGCGGCGCACCACAGCGCCAGAGGCACAGCGGCATGGGTGATGATCGAAG	2	40324
GAGGCTGGCCGGAACCGCGCTCTTGTCCAGATAGCCCACCGCCTTGGTGG	2	40324
TCGGCGATGCCGAGGCCGGCACCCTGCAGGCCAAGCCCGGTATCTCCATC	1	40324
GAACGACATCGTGCACCTCCTTCAGCGGATGCTGCTGTTGCGCGGCCAGC	1	40324
GCCTTTCATCTCCACGCAACCTCAGCCGCGTGGCGGCGTCGGATCGGGGT	2	40324
GCTCGGGCGTGCGCACGTTCACTTCGGCCACGTCGTCGCGGTCGCGCAGC	1	40324
GGCTACTACCAGTCCAGCTTCAGCAATGAAACGCTGGCCGACGGCAGCAG	1	40324
TTACCCCTACACCCAGTCGAAGGAAGTTAGCCCGCTGACCAGCTCGCAGA	1	40324
TTACACCTACACCCAGTCGAAGGAAGTTAGCCCGCTGACCAGCTCGCAGA	1	40324
ATCGGCCGCGAACTGACCGGCATCAATGCGTTCTGAGTGCACCGGTAGTG	1	40324
CGACAGGCCCCGCTTCGGCGCAAAGCGCCGAGCCACTTGCGACTTAATCC	1	40324
GGGCGTGCAGCAGACACTGGACATCCTGCGCGCCTGCCGCGACAGCGGCA	3	40324
TTCCACGAAGAGAAGCCGGCGTTGATGGGCGAAAAGCCGCTGTCCTTTGC	1	40324
CCACCAGCTGCGGGGATCTGTCAGAGGTGGGGCGGTGTCGGAGTGCGGGG	6	40324
ACTGGATCGGCACCCCGTTGGGCATCCGGCCGCTCTGTGCCGCGCTGCCC	7	40324
CCCCAATACCGGTGATGGCCGCGCCGACCACCTGTATTACGCGTTGTGGG	1	40324
TCAGGTCACCGAACCCGTTGTAGGCATAGGTCGTATGCAGGCCTTTCGGG	1	40324
CCGATGCCAAACGTCGGGGAAACGCCGCTGGCGGTGGCTGGGGCTACGTC	1	40324
ACGTCAACCACCACCAGGGCCAGGAAACGGACAACAAGGGCACGGGACGC	1	40324
GTCAACCACCACCAGGGCCAGGAAACGGACAACAAGGGCACGGTACGCAG	2	40324
GCTGGAGATGGTGCGCAGCATCCGTGGTTTCGAAAACGCGCACATCACCC	2	40324
GCTGCAGGTTGCGCCGCAGATCGACCGCGTCCTCAGCGACACCGGCCGCA	5	40324
CGACAACGACATCCGCACCCTGCTGGCCGAGCAGCTCGGCCGCGCCGGCT	1	40324
GCGCAGCCCTGCCGAACCAACCCTACCGCCCCGGCAACTGCGCGAACGCG	1	40324
GTATAAGTGGATGCCCACGCCGGCATGCGCATCGGGCGAGCCGTTGCCAC	1	40324
AGACCTGGCCGGACAGCACCGATCCGCAGCATCCTGCGGTGGGCTTCGAT	1	40324
CGCGCAGCGTGCGCAACCTGCGCATGTCCGACCGCCAGGTGACCTCGTAC	1	40324
GACCAATGGCAAAGCCGAGCGCTTCATCCAAACCAGCCTGCGCGAATGGG	6	40324
CCGCGGTGCCACATGCCCGGGCGACAGCCCCCATTCGGCGAACAGCTGCT	3	40324
CGCACTGTCCTGATGCTCTCGGCCTGCCTGCTGCTGGCTGCCTGTGCCTC	3	40324
AACAAGACAAAAGAGCAAGGATCAGCCACATCAGCAGATTGGCTTGGGAC	3	40324
CGCATCAACAACACGCTGCTGCGTGCCGATCAGCTGCACCACGCCGAAGG	2	40324
TACTGGAAGTGGGTGCCAGCGCCAGCGGGCAGGCCTCGGCCGGCACGCTC	1	40324
TGCTGGAAGTGGGTGCCAGCGCCAGCGGGCAGGCCTCGGCCGGCACGCTC	3	40324
TGGGCCAGACCACCCACGTCTACGCGAACTGGGGCCGCACCTTCCAGGTG	3	40324
GGCCGCGGCATACGGGTGCGGACGATGCGCGCCGCTGGCCAGCACCAGCT	1	40324
CTGGCCGTAGCCGAGCGAACGCATCCATTCGGTCCGGGCCCGTTCCATGA	7	40324
CCAGCAGCACAATGCCGGCCTCGTCAAAACCGTCGCCGATCGCCAGCAGC	4	40324
CACTGAAACCGGCGGTGGCCTGGATCGAGAGCACGCCGTCGTCCTTCACC	1	40324
GCGTGATGCCCAGCATCTGCAGCTTCCGCAGCGTGTGCCGGCGTGCATCG	3	40324
GGTCATGCGCTCGCTGTCGAGGCGATTGGTGTGCGAGTACGAAAGATCGG	2	40324
GCTATGCGCTGGTCAACAACATCGCCCCGGCGCACCTGGAACGCATGGGC	1	40324
TCGAGGCCCGCGCCAAGGGCCGTACCTCCGAAGCGATCAAGCGTCTGGTC	5	40324
ATCATCGACACGCCGGGCGGCAGCCCCTTGCCGTCGTAGTATTCGCAGCT	1	40324
CCACAGTCCGGTCTGGGCCTCGGTCAATGCCACCGGCGTGGCCAGCGCAG	2	40324
GCCGTGCCTACAGCAACTGGTATGGCTTCGGCGTAGTCAACGCTGCACGT	2	40324
AGCTTCAATCCGCGCTCGGCCAGCCGCTTCTCAAGCCCACGCATCTGGAT	1	40324
GCTGGAACGCGTGCTGCCGCAACTGCACGCAGGTGGCTTCGATCCGCCGT	1	40324
TTCCCATCCTGTTCGCGCTGATCGGTGGCTTCGGTGCCGGCATCGACGAG	1	40324
ACCCACTCGAATCCGTCGCCTGCTGGCCGGGGAAGCGCAACGCCAGCTCG	6	40324
GTACCCGCGCTGGGGCGGCAGCCACGAGGCGATGGAGGATTTCATCGAGG	1	40324
TCACCCAGCTGCGCATGGGCCGGTGCCAGCAGTGCATCGGACAACGTCTC	3	40324
CTGGATCTGGCCGACGATCTGGGTCAACTGCTGCACGGTGCTGTTGGCAT	1	40324
GCACCCCGACGCAAGGCGGGGTGAGCCATGTCGTTGCACCAGTATCGGCG	2	40324
CTATAAAGCGTGCGGACCGGGGTCCGCACCCACCGGAAAGACCCTTTAGG	3	40324
CAGGCAGACGTGCCGGTGGTCGGCTTCGTCAACGAGAACAAGCTTGAGAT	10	40324
GTCTACGCCCCGTCCCCATGACTTTCTGCACAGGGAGTCGGTGTTATACC	2	40324
GTCCACCACCCGCGCGTTGACGATCACGCGGCTGTTGCCCGGCTCGGGCA	1	40324
GGCCGATGCCGGCAAGCAGTTCGATGACACCGCGATGGCCTGGTTGCAGC	1	40324
GCCAGCCGAGCAGCGTTACCAGCGGCAGCTGCAGGAAGCTGATCGGGGTC	3	40324
GATGGTCATCCGCCCCAACAGGATCGAACCGGTCCGGATGTGCGAACGGG	2	40324
GATGGTCACCCGCCCCAACAGGATCGAACCGGTCCGGATGTGCGAACGGG	1	40324
CCCCCGCCCCTTCAACGGCCGATGCCCCGCAACGACTGACCTCTCTCGCG	2	40324
ACGGCGATGGCAAATACGCCGCTGACCTCACCGTCGCCATCGCGCAGGGG	1	40324
GACCAGCACCGCTCCGCGCGGAAACTGCACCGCAGTGTGCCCTTCGAATC	1	40324
AGCATGACTGGGTGGACGCCTATGCCAAGGCCGTGGCCAAGGGACAGGAC	1	40324
CAGGGTGACCACCTGGTTGGTGAACGGTGCGGTGGCGACCACGCCCAGGG	1	40324
GCCGCGCATCCGGCCAGGATTTCGCCGCCTTCCTGCAACAACGCATCTTC	1	40324
CAGGACTCTGGCGAGGCCTGCTGCCCACGCTCTGCTTCCTGGTCGCGATC	2	40324
TCGATGACCAGATGGCCAGGGCGCCAGCGCAGCGTCGCCTGTGCCTGTAC	5	40324
CGATGACCAGATGGCCAGGGCGCCAGCGCAGCGTCGCCTGTGCCTGTACC	5	40324
TCTTGATACAGCTCGATCTTAGGGCGGCTGGGTCCGGCTAACAAGCCCGC	1	40324
GGTTTCCAGGCGCAGGTCGAAGATGCTCATGCCGACTCCATGTCGAAGGG	3	40324
TTCGTCGCCGTCGAAGGCGGCTTCAAGGCGCAGCCGGTGCTGGCTGGGCG	1	40324
GCCGTGATCCGGCACAGCCACGCGAGCTGAGCGAAGGCGCGCAGATGGTG	2	40324
CCACCTGATCACGCGACTCGGGCCACGGCACCGATGGATCATCGATGGCA	1	40324
AGCCGTCCACGGCCTCCATCGTCTCGGGCAACCTCGGCGTCAGCGACGAG	1	40324
CGGCATCGCCTCGGCGGTGTTCCGCCTGCCTGGCCTGTACGGGCCGGGGC	1	40324
GTCCGGCGACAACCGTGGCCTGGACGTGCCGTTCTACTCGCGCGACACCG	2	40324
TGATCACGCCGCGCAGCGAAGCCTATACGCCCGACCAGTTGATGCAGGCG	1	40324
AGCGCTCAAGCACCCACAACCTGCTGCCCGCCGTGGCGCCGACCTATACC	2	40324
TCGAACCCAGCGCGCTGGCGCAGTGGCTGCAGGCCGATGCTGGCGGGCTC	1	40324
GCGTAGTGTCGCAGCCCTCGGGCTGGCCGGCCTGCTGGCCTCTGCCCTCC	3	40324
GCGGATCGTTGGCAGACGTCATGCCGGAAGTCTAACCGGCACGGCATGAG	3	40324
CTGCGTGGCAGCCGGCTGGCTGTCCTTCCAGGCGGCGAACTGCTCGCGGT	6	40324
CCCTTGGCTCAGCGCTTGCCGCCCAGCAGGCTGCCGCCGATCTTCAGCAG	1	40324
GGGCATCGCCCAGCACCGGCAGGGCGTTGAGGATGCCACCGCCGGTCTCC	1	40324
GAAAACCGTCCCCTTCCCCATGAGCATTCCATCCATGCGTAGTGTCGCAG	1	40324
GTAACCACCATTTTCCAAAGATGTCCCGGATGAACAACGTACAGAGAGAT	12	40324
GATCCGCACCCACAGGTCCGCACCCACAGCGGATAGGCAGGAATGCTAGG	1	40324
GAGATGATCAGGCGTCCTGCATCGCGGCCGACCGTGTATCCGTTATCGAC	3	40324
CGCTTGCGCCGCAGCACCCACTGCGCCTGCGACAGCAGGCGTGCATCCAG	1	40324
CATAGTCTGCCACTTCGTCCCTGTTTCTGGTTCCGCCACCTGCGTCCATC	2	40324
CGGAATGGGCGACGCTCGCCGTCGAAGTGCTGGCCGAGAGCGCACGCAAT	1	40324
CACCTCCAGTTCTTCCGGCTCGTCGCCGGCCAGCTTTTCCGGATACAGGT	1	40324
CAGGGGTCAGATCCGTTTCCTCCGGGAAACGGATCTGACCCCTTCCATGT	1	40324
GTTCGCCGCTGCCGTGGTCGAAGCGGATCCTGCTGGCCATGCTGGCCGGG	4	40324
GGCCAACTGACCGCGCAACGCAGCGGTCCACGGCCGCCGCCGGGTCCTGA	2	40324
GGCCACGTCATCGCCCAGCACCGTCTTCAGTCGCTCGACCAGGCCCTGCT	1	40324
CGCCTGCACCACGCGCCGTTCGCTGTCGACGAAGTCATCCACGTGCTGCA	2	40324
CACCCCCACCACCTTGGATTGCGGCAGCAGCACCATGTAGCGCGACGGAA	1	40324
CACCAGACGCCGCATGCCACACCTGCTCCGGTAATCGAGCAAATCAAGGA	1	40324
TGCTGGCCGGGCAGCAGTCGCTGGCGGCGGCGGTGCGTGGTGATCTGGAA	1	40324
GAAGTTCACCGAGCATGGAAAGGTCAGCCTGCACGTGCGCGCGGCCGGCC	1	40324
GGTGCGTGACAACCTCTACGGCATCGAGCGTGAGTTCGCCGGCCTGCAGG	3	40324
CGGCACTGGCCGCACGCTGCGCGGCCGGAATCACGCCGCCGGTGTACAGC	1	40324
AGGCGCACGGCGGCGGCATAGCTGAGATCGACCACGCGGCCGTCATGGAA	2	40324
ACGTAGTCGTTGGCTTCACCCTCCACTTCCAGGTGCAGGCCACCGACATT	1	40324
GTTCCAGGCTCGGGCGCACTTCCACCGAGGCCAGGGTGGTGATCGGCATC	2	40324
GCACCGGGGTCTACCGCGAAGAGCATCCGCGCATCGCGTGGATCTACCTG	3	40324
ATCCTGGAGGAGTGGGCGCGCAGCGGCCGCGACTACGTGCTGTGCGGCGA	1	40324
GAAGGGCTGGGCGCTGCGCAGTGCGTTGGCATGGTCGCGTGGTGACAACC	1	40324
CGCGCTGAGGATGCAGGAGCAGCAGCTGCGCGACATGTCCTTCAAGGATC	1	40324
GTCCAACGACATCGAGACAATTTCCTTCGCCTGCCGCGAAATGATGGCCA	2	40324
GAGGCGGTGCTCGTCGACCACGCCGCTGCCATCGGCGCCGACATCAGTGC	3	40324
CAACTGGGCCAGTTCACCGCGCTGCTGGGCGCTGAAGCGGATCCACTGCA	3	40324
GTGCACGGCATATTGGGGGTCATTGGAATGCGCTCAGTGAAACTCCGGGG	5	40324
AGGGCGAACGCTGCTGGTTCGTCGCGGTCTCGGAGATCCGTCGGCTGGTG	1	40324
CACCCGGTAGTCGCAGCACAGGGCCAGCACGCAGCCACCCGCCGGGGCGT	2	40324
GGCGGAAGCTGCGACGGGTATCGGCCAGGCGCGCCTTGCGCTGTGCCGCC	1	40324
GTCCAGGTGGCCAGCGACGCCAGTGCCAGCGCGATCATCACGGCCTTCAC	2	40324
GGTACCGGTGCTGCACGGCGCACTCAATGGTGACTGGCTGTGGCGCGCCG	1	40324
GATCCATCCGGGTAGGGCGCCATAGTCTGCCACTTCGTCCCTGTTTCTGG	3	40324
CGTATGTGGTGGTGGGGGCGGACTGGCTGGACCAGCGCTCGCGGGTGCAC	1	40324
TAGCCGTCTCAACCTTTCCATCCAGCACCGCGGTCGGTGCGGAGATGATC	2	40324
GGGACCATAGCCCGTGGGGCGCTGTCGGGCAACGCCCGATGGTCAGGGGG	1	40324
TGAGTGCGTTGGAACCGAACCACGAGGAAGACTGGTTCCGCATCTACGGT	2	40324

ZOVA Systems

GTTCAGCACCACGTCGCTGCCCAGGTTGTGCTTCGCCCGCACTTCCTCTT	1	40324
TGGCCATGGGGATCGCTGGGGCGCATGCCGCCGAACCGATCGACATCAAC	1	40324
TGGTCATGGGGATCGCTGGGGCGCATGCCGCCGAACCGATCGACATCAAC	2	40324
ACCTGACCCTGGGCCTGGAGGGCTTCAGCTCGGAACTGAAGGCGAACAAC	1	40324
TAGTAGCCCATTTCGCCGCAGACGATCAGGTCGAAGCGCCCTGGCGGCCA	2	40324
TTACCGCACCCTGCAGGCGCGCATCGGCCAGCTGGCCGGCCTGTTGACCT	2	40324
GCTTCCAGTCCGATGACCACACCTGCTTCGGTAGTCACGCCACCACTGTT	1	40324
ACTATCTGAAGCAGACTCACAAACCACATGGATCTGCCCATACTTCAGCG	1	40324
ACGGTAACGAAGATGGTCGACCATGAAGATGGTCTCTGCGCCCAGCAGGC	3	40324
CGACGATCGCGTGGACGGCCCGGCACGTGGCGTCGGCCTGGCACCGGGCG	1	40324
AGCAGAAGCAGGAGCATCCACGCGTGGCGTGGATCTACCCGGAGCTGTCG	3	40324
ACCAACATGCTGACCCAGGTGCAGGTGGTGCTGGCGCACCCGCGCAGCAA	1	40324
ACACCGAGCCGGACCCGCGCGATGATCTTTCGGGCGTGGACGTGGCGCGC	2	40324
GGAACGTGGGCTGGCGCTGGTGCTGCGCGAGGCTGCGACCAACATCGTGC	1	40324
ACGCGTGGCGGAAGTGCTGCCGCCACACGGACGAGTGCGGTTTGCTGGTG	2	40324
CCGGTCATCCGGCGGCCCGAAGCGGCGCTATGCTGGGCGCATGAGCCGTC	1	40324
CAGCACGCTGCCGCCCGCACGCGTGGTACGCATCAGCCCGGTGTTCGCAC	2	40324
GATTTTCTTTTTTCTTCTCCGTGGCTGGACGCACACGGAAACTGTCAGAG	4	40324
CAAGGGCGTGGCGACCGCGCCGGCCGATCTCGATGCCGCCGCGTTCCGCC	1	40324
CAGCGGATCATCGGCATCAGTCCACAGGCCGAGACGCAACCGCGCCCATG	5	40324
AGACCCTGTCCAGCCTGGACGGCAACCCGAATCACGTGTTCGTGCAGGGT	3	40324
GTACTGACCAAGCAGGCAATCAAGCAAGCTGAGGAAAACGGATATGGTCC	3	40324
GCGAAGCGCAGCAACCTGCCTTCCGGCCGCATCAGCGCGATGGCGATACC	2	40324
AGGCGGCCGTCGACATCGGCCGGCAACTGCTCGAACCAGTCGGCATCATC	2	40324
TCCACGCCTGGCGTGGACCTACCGAATCTTCCTTGTGACATGCATCATCG	1	40324
TGCGCAGCGACCGCTACACCAACACGCCGCCCGATGATGCGTTCCAGAAC	1	40324
AACGAACTGGCCGCGACATCGCGCTACAAGTCCGAGTTCCTGGCCAACAT	3	40324
CGGCGGTCAGCGAGGTCGCGGTGATCGGTATCGCCGACACGAAATGGGGC	2	40324
CCGGCCAGGCCGCCGATCACGCCGAGCAGGGAATTGGGTGACGTCCACAC	1	40324
ACCACTGCACTGGCGGCGCAGGCCTACTTCTGGGCGCGTGTGGTCTATCT	3	40324
GTGCCGAAGCGTACCTGGCACCAGTCCAGCACTGCCAGTCCGTTGACAGC	1	40324
CTACCGGTATCCTCACGCGCGGAGAACCGTGCCCCGTAGTAACCCTGAAG	4	40324
GGCATCGCACCGGAGTTCCTCAACCACCTGTTCAGCCGCTTCCGCCAGGC	1	40324
CAATGCGTGCATCCCCGCTGCTCCGGCCGATCTTCACCTCCGTTTTTGCC	2	40324
CCATTGTAAACCGGGCGCCGGGGTCGGATCCCCGCAGGGGCTCTGACCCC	12	40324
CTACACGCTCGGCACGCAGGTGTCCCGCCAGGATGCTGCCGGTGGCGAAC	1	40324
ATCCAGCAGGCCGAGGAAAAAAAGAAGGAAGACAAGCAGCTGCAGCGTGA	1	40324
GCGCTGGTGGACCTGAGCCTGCGCATGGCCGGCCTGGGATGGCGCAACGT	1	40324
CAGGGCGCCCAGCTTCCAGCCGAAGAACAGCCAGCTCCACAGCGCATTGA	1	40324
AAACCATCGGCCTTGTGCACGGCATTGTCCGAACCGCGATACTGGCCATC	4	40324
CAAGCACCACCGGCAGTGGTTCAGGGACATCCTGCGCGGACGTCGGGAAG	2	40324
GTGGGTGAAGCGGCCTACAACGGGGTGAGCATTCTCGCCACGCAGTAAGC	1	40324
GGCCTTGTACGACTTCATTTCGCCGGATATCAGCGCACGTGCGAAGTCGA	5	40324
GACGGCGACCAGCTCGATGCCACGGTTGGTCGGCTTCAGCGCAGTATCGT	1	40324
TGGTGCTGGTGAAGCTGCGCAGGAAGTTGCGTTCGTTGCGGTCCAGGCGG	1	40324
GCACACCCTCATGATCCGCCGCCAACAGCAGCCGGCCAGCGGCCGGCTCT	1	40324
TGGTAGGGGGCCCCCCGCCATTGCTGCTGCGCGGCCGCATAGGCCGGATC	1	40324
TGGTAGCGGGCCACCCGCCATTGCTGCTGCGCGGCCGCATAGGCCGGATC	1	40324
CAGCCTGATCTGACCGGCGCGGCTCGGACAGGGCCAGCAGCAGGGCAACC	2	40324
CGGCGACGATCGGGGAGACACGCGCAACGCATGCCCTGCCACTGATAGAC	1	40324
GGCCTGGATACCTGGACCCGCTTCCAGGTGTTCACCCCCACTGGCGATGG	1	40324
CGTCCCGGATCTGCCTGCGCCAGCCGCAGGCCAGCTGACATCCGGCAATG	1	40324
GTTCAACATCAACCGCCAGGAGATCTCGCGGCTGCCGGTGGCGCAGGACC	1	40324
CTGATCGACAACCTGGAGTTCTGAGCCGCATGCGGCCCGGGGGCGCCCAC	1	40324
TTCGCCGCCGCGCAGCTGCCGCGGTGACACGTTCACCGACACGAACAGGT	1	40324
GGTGGCATCACCGACGCCACGGCGGTGCTGGTCAAGCTGATCGACGGGGA	1	40324
CCCATCGTTGATGCTGCCGTAGACGTTGATGTTGCCTTCGGCCCGCAGCA	1	40324
CATGTCGATCGCGCCGTAGACGATGCCACCGCCACTCCAGTTGAAGGTGT	3	40324
GTACACGTGCGCCCGTTCGTGATCATCCAGCCGGATCAGCGGCTCCACGC	3	40324
CTCCGACACCGCCCCGCCAACCCACGGAATGCCGCTTCTGCTGTTGCTGT	12	40324
ACAGTGCACGCAGGCCCCAGTCGGCAAAACGCGCCAGGTGCAGGCCGTAC	1	40324
GTAACCCATCGCGGCGCCGGTGGTGATCGCCTGTGGCGATGACGACCAAG	2	40324
AGCATGTCCATCGCAGTCAGCGCCGCCATCACGGCCGCGACCAGCAGCAT	4	40324
GGCTCGAAGCGCTCGCGATCAAGCGCATCGAGGATGTTCTTCGCCGACTG	2	40324
GAACAAGGGCCAGCTCGACGCCGTTGCCAGTGTCGCCGACAAGACCAGCC	1	40324
ATCAGAATCATTGCGCCTTACGGGCACTTCACCGCATACACCGGCCCAGC	2	40324
CCAACTTCCGCACGCTGGATATCGACGATCTGCTCGGTGAGGGCCGTGAC	2	40324
GAAGTGCTCTACGACAAGGACCGCAAGCGTGCGCGTGGCGTGGAGGTGAT	1	40324
GTAACGGTGGTCGCCAGCGGCAGCCGCATGCGCCTGGATGACGTCCAGGC	2	40324

CCTTGCGTGCGGCCGCGATTTCCTTGCGTGCGGCCAGCAGGTCCTTGTTG	2	40324
GAACGACGGGCCCAGCGCGTTCTTCAGTGCCAGCAGGTTGATGTTGCCGG	1	40324
AGCGCCGCAGTGCCGGCCAGGATCTGGCCGCGATCTATCGCCTGGAGTAC	2	40324
TGAGGCCTGTGCGGACCATTGCGGCTGACCTTCGGCCGCAATGGTTACGC	6	40324
TGCCAGTGCGCGCTCGTCGGCGGCAGGCACATCGCCATAGGCCTTGAGGA	1	40324
TGTTCACACTGTTCGACAGCATGCCGCTACCGCTGTTGCTGTCGTGCATC	2	40324
GCGCGGTGCGTCGTCGAGGTAATGGCCCTTGTTGTCGCGGTAACGCAGCC	1	40324
GTCGAGCACACGCTCACCCTGCGCCTCGCGCTCGAACATCACCTTCATCA	1	40324
GGTGAAGGACGCGCAGCGCCTGGCCGGCGCCGAGCAGCTCGATGCCGCCG	1	40324
TCTACGCCAAGGCGCTGGGTGGCCAGGTCACCTCGGAAATGAAGTACCGC	3	40324
GGTACACGCATCGGCGAAGCCATCACCCTGCTCGGCGAACTGAGCCGCAG	2	40324
ACCTGATGTTCATCGGTGCCGCTGTTGGCGGGCGCTGGGGTCGCGAGAAT	4	40324
CCCCACTGGTCAGTGACCCCGGCTTCCGCCTGGTGCGTGCTGCCCGTGCG	3	40324
CTGGAAGCAGCGCGCCTGGCTGCAGGTCGCCCATTCCAACGATGCGTTGG	1	40324
GCTCGGCTGGGCGGTCAGCGGCGCCGGCATGCTGATGCTGGCCTTCGTGT	4	40324
AAGTACCGCGACATGCCGCCCTCCGACGGCTCGCCGGGCTGCAACGAAAT	2	40324
TGGGACCGATGGCCAACCCGTTCTACGTCGATCTCGGCCTGGACGAGGAC	1	40324
GGCCCGAAGGTCCTGCGACCCCTGTGCGAGCGCGGCGGGATCGTCTAGGG	1	40324
CTCCCTGCAGGCAGTGCTGCGCCCCGGTGCGTTGTTGGCAACCCTGGCCC	1	40324
CTGGCCATCGGCATCCATGAACAGCGTCGGCCCCAACAGTGTGTGGTACA	2	40324
CATTTCGGCCACGCTGTTGTCCCACGCCAGCAGCTTGGCCAGGCCCGAGG	1	40324
GTTGGTGAGCACCTGCAGCACCAGCGCCAGGCCCCCCATCAGGATGTAGA	2	40324
CCGCACACCGCCACCGCGGTGACCGCCAGCGAGTGGACCCACGCCTACCC	1	40324
GCTCACCACCAGGGCGCCTGCCAGCAGGGCAATCACGAGTGCGCGTTTCA	1	40324
CCGGCGACCCTGCATTGATGCCGCGATGCAGGCGCAGTTGCAGCGAGCTG	1	40324
TGGTAGTCGTGTTCGCGGACCTTGCCCATGTCCACGTAGCGGTAATGCAT	3	40324
GGCGCAGACCGTGATGCAGAAATCCCGCCACGTGATGATGGTCGGGCAGG	1	40324
CTTCCGCCTGCACCATGTCGGTTTCGTGTTCCAGGGCTTCAATCTGTTCC	4	40324
GCCATGCTGCGCACGTTGCGGTTGTCCAGCAGGGGCGGCAGCCGCCGCGT	2	40324
GTATAGCTACAACAGATTGTCTGATATCGAGAAGCAGAAGTACTTTGCCA	1	40324
AGCTACAACAGATTGTCTGATATCGAGAAGCAGAAGTACTTTGCCATAGA	1	40324
CATCGACGTCAAGACCGGCGGCACGCTGACCATCGAATCGCTGCAGGACA	1	40324
CGCCGATCTCGACCAGCAGGTGATGGACCATGCACTGGGCCTTCTCGACC	1	40324
CTACCCCGGAAGAGATCGCTGGCACCGTCGCGGTGATGGGTGGCGAAGAC	1	40324
AACACCAGGCCCACGCGCAGGCCGGCGAAGCGCTGCGGCTGCGCCAGCAC	1	40324
GCGGTCGCGCAGGTTCTCGCTTTCGCAAACAATGCCGGCTTGTCCTGGAT	2	40324
ACCCACCTCAATGCCCGTGGCCAGCTGACCAACCGCGGCCTGATCGATGG	1	40324
CCCCACCAGATCTCCGAGGCGTTGCCGCCGTAATTGACCACCTGCACCTG	1	40324
CCCTTGATGCTGGCCGGGGTCATGTCGAGCTTGAGCGAGAACTGGTCATA	2	40324
GACCTGGCCGTCCCTTCCATGCAACCGCGCAAGACCGATCTCGCCCGAAC	1	40324
GCTCCAGGCTCGCATCCAGGTCATCGACGCCGATGTAGACGCGCGCCCCC	4	40324
GCGCCAGACCTGGATGAAGCGGGCCTGGCCCACCCAGCGCTCCACGCCGT	1	40324
GACGACGCCAGCACGCGCCAGGCCGAACTGCGCAGCGAGCTGTTCCGCAT	1	40324
ACCGCTGACCACAGAGGATGGATTCCATGAAACGCTCGCTGCGGATCCTG	2	40324
GCGTCTGCCGTGCATGCACGTCCAGCGCGGCCGAGCCGGTGGGGAACAGG	3	40324
CTGGGACCAGCACAAGCAGGAACTGCCCCACTTCCAGGATGTACGTGAAC	1	40324
GAGTTCTACGACCTGCTGGCCGAGGACCGGAAGTTGACCCTGCGCCGCGA	1	40324
CATCTCGGGGTCGTCGAACGATTCCGGCGTCACCTTCACCAGTACCTGGG	1	40324
GACTACGCGCGGCTGAGGCGCGCCTATGACATGGCCTGGAACCGGGGCAC	1	40324
GTCTGGGCACGCGCGAGATCGAGGAGGCCATTTCCAGCCATCCGCGGGTG	2	40324
CGGTGACCAGGTGACCGTGGTCAGCAGCAGCACCGGCCTGACCCGTACCG	1	40324
GCTGATGTAGGTGCCGCCGACGGCTTCGCCCGAGGACAGCGGCTGGGCAC	1	40324
GTCTTCGCCGCCTCGGCATTCCACGCCGCCACCTTGGCCTTGGTATCAGC	1	40324
GGGCGTCGCCGAGGGTGTCATGGCTGGCGTGCCACTGTTCCAGCGTGCGC	1	40324
GTTCTCCCTCGCGCCGGCGGACCCGCCGGCGCCGACATTGAAGTGCCAGC	1	40324
GTCCTTGTTGCTGCCGGCCTCTGCCCTGCTGCTGTCGGCCTGTGTCAGTA	1	40324
CGCTGCCCGAAGTAGGCCTGGGCCAGCTGCACGGTCAGCGATTGACGTTG	1	40324
GCCGGCATCCAGATCCTGCGCCAGCAGCGCGGGCACGCGCAGGCCGTTGT	2	40324
GTCTATGACGAAGCGCGCCTGGCCGCGCTGAAGAAGGCCCAGGCCCGCGC	2	40324
CAAAGCCGGTGATGCCCGGGAACAACTGGTCCGGTCGCGGGGTCTTGTAG	1	40324
GGTACTCCTCCAGCTGGACGCGGCGGGCACGGGCCGTGCAACCCAGTACC	1	40324
GCCTGGCGCTGGCCGTACAGCCAGGCCGACAGGCCGGTCAACAGGACGAA	2	40324
GCGTGCCACCAGCCCCACCGTCGCTGCGGTACGGCCACGCGGGGCCGGCA	1	40324
GCGCGCTCTGGCGCAGGAACACTTCCGCGCCCAGGCGGGCGGTCATGTCG	1	40324
GCGGCGATCAGGTCGGCCACTTCGCCGGTCGGCGAGGCCACCAGCTGCGC	1	40324
GACGGTGCTGATGCGCCCGGCCTCGTTCACTGCGGTTGGTGCCTCGGCGT	3	40324
TCAAACCGATGAAGCGGTGCTGCGGCGGGTCACCCCGCGCTGGCGCACGC	1	40324
CCGTCGCCACCATCCACAGCGGTCTGTGCCTCGCGGTCGCTGACCCGCTC	1	40324
AGCGCTCCGGTAACGTCCACCCGGCGATTGAACGCGGCAGAGCCCGAGAC	1	40324

GTCACGCCTGCGCATCCTGCTCTCCGATCGCGAAGCCGGCGCCGTGCTGG	1	40324
GCCTGCAGGCGCTGCCGGAGACGGTGCGCGCTGATGAGTTCGTACTGGTC	1	40324
ACTTCATTGACCGTATGACCGACAAGGGCCCAGTGCAGTCCGTACTGGAC	4	40324
GTCCAGCAGCGGCGAGTTGCCCAGCAATGCCAGTGCCAGCACCGCCCAGC	1	40324
GGCCGTGGCCAGGCGCGGATCCGCCTGGCGCTGGCCGTACAGCCAGGCCG	1	40324
GGCCGTGGCCCGGCGCGGATCCGCCTGGCGCTGGCCGTACAGCCAGGCCG	1	40324
AACACGCTGTCGAGCGGGCCATCATGGCCGAATGCACGTACGCGTTCGCC	1	40324
AGCGCGCGCGCCAGTACCTGCCAGATCCTCGGGTCCTGGCACTGCGAAAC	2	40324
CGGCGGTGTTGAAGTTCGCCAGCGGCGCGGCCAGTGCGGCGGCATCGGCC	1	40324
GACGTGCACCACGAGGCCAGCGCGCGCCTTCGCGCACGCCCGGAAGCCGG	2	40324
GCCGTGTCGGATGCGCTGGACGACGGACGTGTGGTGTCCGGCGTGGGTGG	1	40324
GCGCCTGCTGACGCCGACGAAGGCGTGGTGCAGCTGACCCCGGAGCAGAT	2	40324
TGGCTCGACGCTACAACGGGTACGGCGGTAGCGATGATGCGCAGCGAAAG	1	40324
TGTCACGACATTGGCCGGCAGTGCCGATCCTGGTGCTGTCCGCACACTGC	1	40324
CGAAGAAAAGCGCTCCACCACCGCGCCGATGTTGCCGAACGGCGCCACCA	1	40324
GTACATGTTGCGGAACAGCTTGCGGTAGCCGGCACCGTGCAGTTGCGCCT	2	40324
CATGGCACCGTGTCGCCGTCGCAGGAGGGGATGGCATGGGCATCGGCAAC	1	40324
GATGTAGCCGGCCGGTTCGGACAGCAGGAACGCCACCGTGGCCGCCACTT	2	40324
CAGCCCGATCGGCGCGTTGTCCAGGATGGTGCGGCTGAAGGCTTCACTTT	2	40324
GGACCAGATGCTGGTGCTGTTCGGCTGGCCCGAAGCGATCGATGCTGATC	1	40324
CTCATATCCTCAGGCGGCTTGGACGAACGGGTCTTTTTCATCGGCTTTCG	1	40324
TCGGCGACATCGGCGTACCGTGGCAGTGGGAGTACGGCGCAGCGAAGGCA	2	40324
CCCACGTACCGGTGACGGACCTGGCCGAGCGTTTCGACTGGACCATTGCT	1	40324
GGGTGGGGGAAGCAGCTGGGCAGGTGCGATGCTGAGGTCAGCGCGGCAGG	1	40324
GTACAGCGCGTACGACTTCCCGGGCGTGAAGGGTGCCAAGGTCAACGGCA	1	40324
GCTGGCGTGGGTACAACGGCCCGACGGTCGCCGCATCAAGTATCTCAAGG	3	40324
CTGGTATGCCCGCACCGCAAGCAGTTCTCGCCCGCGGTACGGCAACTGCA	1	40324
GTGCAGGCGCCAGTCGTCGCTGCTGCTGTCAACGTCGCGACCATCATGGA	1	40324
CTCCCATCGCTTTCCACGTCGGGTGTGGAAAACCGGCGAACCTGATGCAG	2	40324
TCCACCAGCAGCGAGCCGTCTTCGCGGGTCACCACCAGCGCGTCCTCGTC	1	40324
GGGTGATGATCACCTCGCAGAACCACGGCTTCGCGATCGATGAAGCGACC	1	40324
TGATTATAGTCGGGGTGGTCAAAGACACCGTCTGTGTCGCGTCTGGCCTT	2	40324
GCTCTACCCACCACAGCTGGAACGCGTGTTCGCACGCACGCTGGGCGTGC	1	40324
CCGGTCGGGTCCAGCCTCCTGCCGGATCCGTCGACGCATGCGAACGCTGT	1	40324
AAAGAGTACCGCCCTGATCACCGCCTCCGGCCATGCCCGGCTGAAGGCCG	1	40324
TCTACGCAGTAGCCGCTGCCGGCGCGCGCCACTTCGCAGTTCGGGAACGT	3	40324
GATTCGGCGGTCCTGGTGCTGGCCAGCATGTCCACCGACGAGGCTGGCGA	1	40324
CCTACCACAAGGTCAACGCGCTGTTCGCCGACAAGCTCGCGCCACTGCTG	1	40324
GGCTGGCGGCCTGTTCGCGGAAGCCCGGCTCGGCGTCGGCGGCCGGCTGC	1	40324
GTTCACTGCGGTTGGTGCCTCGGCGTGGTGGGCGCTGGGCTATGTGGCAG	2	40324
CTTCAGAGACCTGCAGAAATGCAGGTCTTTTTTTTGCGTGCGGCCTGCGA	2	40324
CCCTACCTACCGCTCGTCAGCCCGTCTGCGCATTTTGACACCGGGCTGAC	3	40324
TTGGCAGCGCCGGGTCGGCCGCGGCCTCGGTATCCAGCGGATGCCTGATC	1	40324
CTTCGCGCCGCTGGGCTTCACCGTCGCCGAAGAGACCATGGCGCTGATGC	4	40324
GACGAGGATTTTCATCTGGTACGGGGTCCTTCAATAACGGGGAGAACGTC	1	40324
CTTCATAGCTGATGAAGATCTTCTGCCCGGCGTTGTACAGCCACGGGCTC	1	40324
CAAGCACATTGTGCCGGTGGTCGCCACCTGTGCGGCCATCGATGCGATCT	4	40324
CAGCTGGTCGCCAAGGGTGTCCAGCAGCTGCTCGTACAGGTACTTCTCGC	1	40324
GGAGGAAAGCATGGCGGGCAGGATACGGCCGGCCGCAGATGCTACGAACC	1	40324
GCAGGTGCCGCCGGGCGGCCGAGTGCGTTGAGTGCGGCCGCGTCATTGCC	1	40324
CATCCAGCGCGCGCGGCGTACGTCGCGCACGTCACCGCACTCGACCACGG	1	40324
CGGATGGCCGGATACAGCTCTTCCAGCGAGCCGGGCTGGCCCGGAACCGG	2	40324
CGGATGTCCGGATACAGCTCTTCCAGCGAGCCGGGCTGGCCCGGAACCGG	1	40324
CGGAGTGCTCATGCGACCTCCCGGACCAGCGAAAGCGGATGGCGCTGGCC	1	40324
AAGCAGCAGGGTCGCCAGCGTGACGATGGCGATGAGCAGATACTTCTTAC	6	40324
ATGCCGAAGCGTGTCAGCAGCAGGCGGCGATGCAGGCGCATGCATACCGC	1	40324
GAAGGTCACCGCGCAGCGCGACCAGGCGACGGCAGCCGATGGCGCGGTAC	2	40324
CACCACCGATGACCACGGTCTGCCCGTCCTCGACAAGCACTGCGGTGTTC	2	40324
GACCAGTACACCGGCCAGAAAGTGGTCGACCAGCAGTTCCACGAGTGCGA	6	40324
CACCATCGGCGAACAACGCGCGCACAACATCCACGTGCATGACGGCGTAG	4	40324
GCCGGCGTATCGCCACGCTAGCGACCCTGCAGCACTCGCCGGATGCGCGT	3	40324
GTGGCGCACTGGTTCGGGCTGCTGGCCGCGAAGAAGGGCCTGAAACACAT	1	40324
ACCGGGTGAGGTGTAGCGGTAGTGCCGGCCGCTGGCCGGCACCCTCATGA	58	40324
TCCAGTTCCTCGGCACTCAAGTGCCAGGTCTCGCCGCTGCAGTACAACGC	1	40324
GATCCCGCGCAGGCCGCGCAGCAGCGCATCGCGGTCCAGGATCTGGCCGG	2	40324
AGTCGGCCGCGCGCAGCGCTTCCTCCTGCTCGATGGTGCCGGCCTTGGCC	1	40324
GGTTGAGCCGCATGAAGTTCTCCGCCCGCTGCGCGATCAGATAATGCGTG	2	40324
GCCCAGTGGTCGGTGCAGGTCGATCAGCAGTGGCCGCAGGTGCAGCGAGG	1	40324
ATGCCGCGCTGTACGACGACCGCCTGCTGTCGAAGGCCTCGCGCCGCGCG	1	40324

TTTCCTTCGGGACTTCCACGCACTGTGCCGGCAGCCGGAATGGGCGACGC	5	40324
TTTCCTTCGGGACTTCCACGCACTGTGCCGGCAGCGGGAATGGGCGACGC	1	40324
CCACCTGACTGGGTGTTCCGGTCAGTGCTGCGGCGGCCGCCGGCCCGGCC	1	40324
AGACGCTGGCGTCGTGTGCCGAAGAACTGCAGCAGCTGGCGCAGACCGTG	2	40324
GCGGTGGACTTCGCCATCACCGGCCACCGCGTGGCCGAGTTGCACAGCGA	1	40324
CCTATGGCGTGATGTGGCCGAACGACTCCATCGCCGGTGGCAGCACGTCC	1	40324
ATTGCTTGCACGTGTGCCGACCAACGGTCGGCACCTACCCGGAATGAAGG	2	40324
GCACCTTGACCGTGTTGCTGAAGCTGATATGGCTGTCATAGACCTCGGGC	4	40324
GCACCTTGACCGTGTTGCTGAAGCTGATATGGCTGTCATAGACCCCGGGC	1	40324
CGCTGCCATTGGTCACCACCTTGGTCTCGTTCACCCAGGCGCCCTGGATG	1	40324
TCCTGCACCCTTGCCGGGGCCGGTGTCAGCATCCGGGCCATGGCGTCGAA	1	40324
CGCAAAACCCCGCGCTGCGCGCGACAGTCGACTAACCGTCGACTCTACCC	6	40324
GTTTCCGCCTGGCAGCGCAGGCAGTGGTGGGCTTCGGTCGGGCCGACCAC	3	40324
CAGTTCGCTGGTGACGAACGATGCGACCAGTACCGGGAACGGGATCGCGT	3	40324
CTTCAGTTCGCTGGTGACGAACGATGCGACCAGTACCGGGAACGGGATCG	1	40324
GGTTCTCGTCGGCATGCGACACGAACACGCCCACCGAACCCAGGGTGACC	3	40324
ACGGCCGCAGCCGACCAGCAGGGCCAGCGCCAGCAAGGCCGGCAGCACGC	2	40324
GAGGTCCTCTTCACCCGGATCGCCAAGGCGCTGTCTGCCGAGTTCGGCAC	1	40324
CAGGTAGTACAACCGCACCTCGCTGTCCACGCGCCGGCCGAACACGATGC	2	40324
GGATGACGCCCACGACCGCCTCTACGTGGGCAACACCGTGGACCTGTCGG	1	40324
CTTCTTCGAAAAGCACGTGCCGATCATCGCCACCGCACTGGTATCCATGT	1	40324
GCACCGACGATCGGCACCGCCTGTGCGGCGAATTTCTCGGTGACCACCAC	2	40324
CGACAAGCATGGGCGCACCGCGCAGCTGGCGCAGTGGGAACTGCTGCATG	7	40324
TCTCTAGGACGAGCTCCTTCAAACGGATTCTTGAATGAGGTGGTGCGCAT	3	40324
GAATACACCAGGCGTTCGGCATGCTGCAGGTCGCAGCCCAACGCCACCGC	2	40324
AAGCAGAAGTACTTTGCCATAGAGATATCGTCTCCTTCGAAAGGAATCGT	4	40324
GCAAGGTACAGCTGGAGGTGGAAGTGCTGGACCTGCGCACGCTGCTTGGC	3	40324
GCCCTGCGTCACATGCTGGAAATCCTTCACCAGCACTTCGGTCACGTAGC	1	40324
ATCTTGCACAGGGGCGACCGGTGCATACCCACGAACTGGTGGTCTCGCTG	1	40324
GCGCCTACGCGCGCGGCTGGCAGCGAGAGTCGAAGAACTACATCGACGAC	1	40324
GCTCAATGTCACCAATCAGCGCGTGGGCGCAGTGGAAACCCGCGTTGGTG	2	40324
GCGCAGGCAGCACAGCCCGATGCTGACCGTCATGCGCAGCGTGGCGGTGC	1	40324
ATGGTGACCTGAAGTACGGCGTGAACTGGTTCTTCGACAATGCCGTGGCC	1	40324
CACGCCCAGGTACAGCTCCGGAGTCAGCAGCCAGCCCGACAGTGGCTTGA	1	40324
TTGCCCGACAGCACGTTGATCAGGAAGCCGCCACTGATGTTGGACGAGCC	2	40324
ACCGGCACATGTTCTACCGGCACGAGCCAGACCTCGACCTGGCCCATGCG	3	40324
GCACCTGCTGGCCGGCGATGTCGTAGGCATAGGGCTCGCTGACCTTGGGC	2	40324
GGTGGTCGGGGCACTGGCTTCCTGTGCGGAAGCAAATGCCGGTGCCAGCG	1	40324
CGCAGACGCTGTCGTCGGTCGGTACCTCGCGCCAGCCAGCGACCTTGCAG	1	40324
AGCACCACGCTGGATGCGGGCACTGCAGTGACGCTCGGCAATGCGGTCAC	1	40324
CAGTGAGAGGATCGACCACGGCAGCGGCGTACCGCCGGTGATCACCAGCG	1	40324
GTGAGCTGCTGGAGCTGCAGCAGCCCTGAGCGCACTCGCCGGGCATGGCC	1	40324
GGCGATGTGTTTCGCCGGCGTGCTGTTGATCGTGCAGGCCGCACTGGTGC	4	40324
GTCGTGGATGCCAGGCCTGCACGATGGCGGCGGTCCGCACCGGGATCGGC	1	40324
ATCTACCTGCGCGTCTGCCCTTCACCGCGCACGACAAATCGTTCGACGGT	2	40324
CTGCTGCATCGAATACAGCTGGCCACCATTGACCGCATCGCGGCTGCCGG	2	40324
ACAGGCGCTTTTCCAGCGCCGCGACCTGGCGCGTGACGCCGGCGGTGGAC	2	40324
GTGCGCTTCCATGATCTGTATGGCGGACCACCCTCGCCGCTGCATGTGGT	1	40324
GTCGGCGACGCTGGCCTGCTCCAGCAGCAGCTCGCCCTGGCGCCATGCAC	1	40324
ACAATGACTGGACGCCGCTGGGCGAACAGGCACGACGCGCCGCGCGACGC	1	40324
GATGCCAGATCGACGGTCGGCATCATCGCCAGGTCGGCCGGCACCTGCGG	1	40324
GCAGCAGTCGCTGCACGTCCGAGAACAGCTTTTCCACCGGCATGCCGTAC	1	40324
CTTGTTGCTGCTGGATCCGCATCCACTGTTGCGGCATGGCGTGCAGGAAC	1	40324
CGATTGGCCCGGGCGATCGAGTTCCGTGATGCCGGCACCAGTGCCTATCT	1	40324
GGGCCAGCAGCACCTCATCGGGAATGCCGATCTTGCCGATGTCATGGAAG	2	40324
GACGGCAGCAGGGTCGGGTCGTGCGACGGCGCGGGCGCGGAGGGGCCGAT	2	40324
CGGGGCGCGCCCTGGGAAGAGACCGGGCGGCTGATCGCCCGCTTTCACCG	1	40324
CCATCTCGCGGGCATGCCGGGCCAGACGCTCGGCCAGGAACACCGACCGG	1	40324
GGGGGATGCCATGCAGCGGCCCACGCAGGCGCCCTTCGCGGCGCTCGCGG	2	40324
GATATAACGCACGATCAGCTGCGAGGCGCGTTCTTTCACCTCGGGCTGCT	4	40324
TCTGGGACATGGGGTGGGATCACCTGGAAAAGGAAAGACCGCCATTGTAG	2	40324
GAAGGCAATACCCCGGCCGCCGTGGCCGCTTCGTCGCTACTTCTCTGGAC	1	40324
GCTTCGTCGCCGGCGTGGTCTGCGTCTGGGGCGTGACCGGCCTCAAGCGC	6	40324
GTTTCACTTCCGCCGCCACCCGCGCGGTCTCGCGCAGTGGTTCGATGCGG	2	40324
GGGGTATCTGGCGCAGGAGTTCACGCCTGCAGCGCCCGATCCCGTTGCCT	1	40324
GCTGCCAGACGCTGCCTTCCGGCAGCGCGTGCGGCGCATGCGCATAGCTG	4	40324
GCCGGGCCGAGGAATTCCCTGAAGGAACCGGCATCGACCAGGCTGGCGAT	1	40324
CCCGGCGTGCACAATGCAGCGGCCACGCACAGCCGCAGCCACAGCAGGCC	3	40324
GTCCCTGGCCTGCAGCCAGGCCCTGGCGGCGGTGCCGGCCAAGGCCCCCG	1	40324
TCCAAGTTCGGCGGGCACGCCTGGGGCGCGCCGGCCGGCATCTTCCTGTC	1	40324
CCCGACCACCGCGCAGCTGCGCGATGCGCTGCCGCTGGCCAACGCGCAGC	1	40324
GCGCTGGCCGAGGCGCGCCGTGCGGCCGAGCAGGCAGCGCTGGCCAAGGC	3	40324
ATCCGGCAGCCAGCCACAGGGCGTAGACACAGGCGATGACAATCGCGCTC	2	40324
CGGGTCTGGCCGTTGACGCGGTGGACTGGCTGCTGCTCACGCACGTGCAC	1	40324
CCTTGGCGCCGATGCCATCGCCACCAACCAGATCGAGATCCATCCCTACC	5	40324
ACTTCGCGGCGTACGAGATCCACATGCAGATGGCCATCGTCGAACACCGG	1	40324
CGTGTGGTCGTTGCCCAACGGCGTTGAGAAGTCGGCCTGCAGGGTGTTGG	1	40324
CCAGGCGACGTCCTGCTGGAGCTTCAGGTCGCCGCGCAGGAAGTTCTGCG	1	40324
CTGGGTATCCAGCGGTGCACTGCCGCCCAGCGACAGCGACAGGCCGTGGC	4	40324
CCAGCCCGGCGTCGAAACCGTACTCGTCCATCAGGTCCAGCGCGGTGGCG	1	40324
GAACAGCGGCGAAACCGGCTGCACCTCGCCACCACTGCGCAGCAGCGTGG	1	40324
CACAGATGGTTGAGGTTGACCACGTCCTGGCCGATCAGGCGCGGGGCGAT	4	40324
CCCAGTCGCGCACCAGCGGAACCTCGCGACCGAGCAGCTCGGACAGGCGC	2	40324
GCACCGGTTGGCTGTGCGTGGCCAACTGTTCGGGCCCGTACAACCAGCGC	3	40324
CACCGAGGTTGCGCCAGGCCAACCGGCTGCGGCCACTGGCGTGCCGCCAC	1	40324
CCCCTGCAGGCCGCGCCACCGGCCGACATGCGCCAGCCGCTGCGGCTGGG	4	40324
GTGGTCGATGCCGTTCCAGTGCTCGTGGCGGGTCCTGGCCGTATCCAGGC	1	40324
TGATGCACCGCGTGGAAGACTGGCGCGAGCGTCTGCTGGCCGAGGGTGAC	5	40324
CGCCCCCTTGAACAACAAGGGGGCTTTCCACCCGATGCGCCGCTGGGAAT	1	40324
GGCGCGCTCACAGTGGGGCCACCGCGCCGCGGCTCGGCGCTTCGCGGCGG	4	40324
GTGCGGAATTCTCCCAGCTGCGAGCGCGCCTCGCCCAGGTGCTGGACCGA	2	40324
GCTCGCGGTCCAGCACGTACAACGGCAGCGCCGCAGCGGTGTCGGCGGTG	2	40324
GGGTGGGAATGATCAGGCGGTCGGCAGCGGCCAGTGCGTTGATCATCAAC	1	40324
ACGCCCTGCCCTCGCAGACCGCCACCCCCGCCGCGCTGGACGAGGTGTAC	2	40324
GCAGCCAGTGTTGCCATCACCGCAGTGAGGCGGCATTGCAGGCGTGGCCT	1	40324
CCCTTGATCAGGAAGTACTCGTAGGCGCCGGCCGCGACCGGCGCGAACAG	1	40324
TCACCCAGCGACGAACGTAAGTAGTCACCAAGATCTAGACGGAGCCCGTT	1	40324
ACCCTGGGTGCCTGGGGCTGGCGTCGTTCGCGCTACGTGGAACTGCCGTT	2	40324
GAACTGGCAGAGACGCCGAGGTTGGCACCGTGGATGCGCCCATGATGGTC	1	40324
CAGCGCGACGGCCGCATCGGCGACTACCTGGTCAAGCAGGGCATCACCAC	3	40324
GGGCAGTAGGCCTTGATGGCGGCTTCAGCCAATCCCTTCTGCGCGCTGCG	1	40324
GCCTGCTGCAGGCGCTGCGCCTGGCCAAGGTGCTTCGCGTTGCACCGGCC	3	40324
CACCATCGCGCCGACCAGCAGCATCATCGCCACACCGCCGAGATAGACCG	1	40324
CGCCGGCACAGAGCGCCAAGTTGTCGGACTGGACCACGATGATCGACACC	2	40324
ACATGCACGCCGCCATAGGAGTCCTGGACCACGCCAGGGGCCTGCAGCAG	1	40324
GTTCATGGCGATGTATACGCCGCTCGGCAGCGACTGCACCGCACCGATCG	3	40324
CACTATGTGGCCGTGCGCGGCGGCTGGTGGAAACGCTGGTGGCGCTGGGC	2	40324
CCGCCACCCGATCGGTCTGTTGGCCGAAGACGGTGATCGGGCTGGCCAGC	1	40324
GTTTGATCGCCAGGCCATGCTTGATGGCGGCGTCGACGCAGCGATGGTCA	2	40324
GCCTACATCATCTTCCTGCAACTGCTGCTGTTCTGGCTGTTGAAGATGAC	1	40324
ATGAACAACGTACAGAGAGATCACAGTTAGTCGACTGCTCTTCGTACAAC	3	40324
GGCCTCGCCACGCAGGTCAGCGCCCTGCTCACGCAGGGTCTCGATATCGC	1	40324
AACAGAACGCTCGGCGCCGCTACCTTACCTGTCGCAGCGGCGCCCTCAGC	2	40324
TCAACGAGGTACCCTCGTTCCAGATCATCGCCCACCGCATCATCTGGAGC	2	40324
GCTCCGAGCGCGAGCGCCGTGCCGATGAAGCCGAGCGCGAGGTCGACGAG	1	40324
AAGGCGGTGACCGCGCACAGGTCAATTCGGTTCACGGGCAGGGCATCGAC	5	40324
GTCCAGCGGCTGCATGCCCTCGGGCATCTTGCCCAGCAGGTAGGCATCGA	1	40324
ATCCGGATTACCTGGCGCGCTTCCCGGTGGCACTGGTCGAAGCCGAGGGC	1	40324
GCCCTCGGTGAACCAGCGCTTGACCGCGCCAATGCCGCGCTCGATGAAGT	2	40324
GGCCGGCGGTGATGCCCGCATGGCCGGTGCGGTGCCGCGCGGTGCCACCG	3	40324
GCCTGTACCTGGTCGCGGTGCTGGCCGGCCTGGTTTCGACCGTGCCCGCC	1	40324
CGTACCGACAGCCGATCGTTCTCGGCCTGCAGGCCATCGATGACCAGATG	1	40324
TGCCATCGGTGATTACTCCACGGCATTGGGTACGCAGAGCAATGCCACGG	1	40324
GGGCAGGCGCAGGCCCTGGAAGTGGTGCTTGCCGCCCAGCGTCAGCGCCA	1	40324
CCCAGGAACAACAGGGTCAGGTAGCCGGAAAACACGAAACCAAGCAGCAG	1	40324
AATACGCGCATCCTGTTGATGTGCAACGACCATACTTCGGCGTGTGGCCC	1	40324
CTGCCTGGGCACAGAGCTCGGGGCAGACCCCTCCAGCCAGGACCCTGTCG	4	40324
ACCTGCGTGCGTCGGCCTCGCTGATCCTGGCCGGCCTGATGGCCAGCGGC	2	40324
CAAACGGCTCGGTGCTGCCGTCGGTGCGCACCAGCTCGACGGTGTACGGC	1	40324
TTTCCAGGGTGCACTGCGGGCAGGCGGGAACAGACGACATCGGCAAGTAC	1	40324
TTTCCGAGGGACGCGAAGGCTTCATATCAGTTGCCGTCCACCATCTCGAA	3	40324
GAGCTGACCTCGCCGTCGGACTCGGACATGATGCTGCGTCCTGATCCGGA	1	40324
TGTTCGCGGTTGAACGCGTGGAGACGGGTCATGAAGCACAATCCTGGAAG	4	40324
GAACGGCGCATGTGGGGCGTACTGCTGGAGTACGTGCTGAGCGTGATCTG	1	40324
CTACGCCGCTGCACGCGCACGCTGCTGCGGAAACGCAGGTGCCGATCGTC	1	40324
TCGGGAAGCTGGGTTACGAAGTACCGAACGTGCGTTGACCGCACGTGGGA	2	40324
CCCAGGTCGACGCCGCCCTGCGCGCGACCCTGCAGAGTGCACCCCTGCAG	2	40324
